# Lattice Engineering in Hydroxyapatite Enables Direct Photocatalytic Synthesis of C_4_ Products from CO_2_

**DOI:** 10.1021/acsami.5c19539

**Published:** 2025-12-16

**Authors:** Marc Arnau, Isabel Teixidó, Pau Turon, Carlos Alemán, Jordi Sans

**Affiliations:** † IMEM-BRT Group, Departament d’Enginyeria Química, EEBE, 16767 Universitat Politècnica de Catalunya - BarcelonaTech, C/Eduard Maristany, 10-14, 08019 Barcelona, Spain; ‡ Barcelona Research Center in Multiscale Science and Engineering, Universitat Politècnica de Catalunya - BarcelonaTech, 08930 Barcelona, Spain; § B. Braun Surgical, S.A.U. Carretera de Terrassa 121, 08191 Rubí, Barcelona, Spain; ∥ Institute for Bioengineering of Catalonia (IBEC), The Barcelona Institute of Science and Technology, Baldiri Reixac 10-12, 08028 Barcelona, Spain

**Keywords:** CO_2_-to-C_3+_, permanently polarized hydroxyapatite, TSP treatment, CO_2_ valorization, enhanced electrical properties, Green Catalysis

## Abstract

Amid the burst of carbon dioxide (CO_2_) capture and conversion technologies, research prioritizing industrially feasible catalysts is vital to minimize climate change effects. In the present work, permanently polarized hydroxyapatite-based biphasic systems have been strategically designed through vacancy engineering and a thermally stimulated polarization (TSP) process, achieving a 15% selectivity toward C_3_–C_4_ products through a single-step CO_2_ continuous-flow reaction (CO_2_-to-C_3+_) under solar light irradiation and mild reaction conditions. To elucidate the underlying catalytic mechanism, extensive experimental characterization has been performed in combination with theoretical density functional theory (DFT) calculations. More specifically, Raman spectroscopy, X-ray diffraction, and high-resolution transmission electron microscopy have been used for structural characterization, and electrochemical impedance spectroscopy studies have been performed to determine charge conduction customization. On the other hand, DFT calculations have been employed to determine the photocatalytic contribution by determining the density of states and band diagrams. The results have been further supported by UV–vis experimental measurements, facilitating the elucidation of the mechanisms behind the photoexcited electrons through band gap trap state generation. Finally, additional adsorption energy studies, combined with Bader charge analysis and Nudge elastic band calculations, have allowed the identification of the binding sites responsible for C_3+_ molecule growth as far as the CO_2_ dissociation pathway and respective energy barrier. These results highlight the role of the crystal lattice vacancies in the CO_2_ bond-cleavage process and represent a huge step toward the design of efficient and scalable catalysts for CO_2_-to-C_3+_ production.

## Graphical abstract

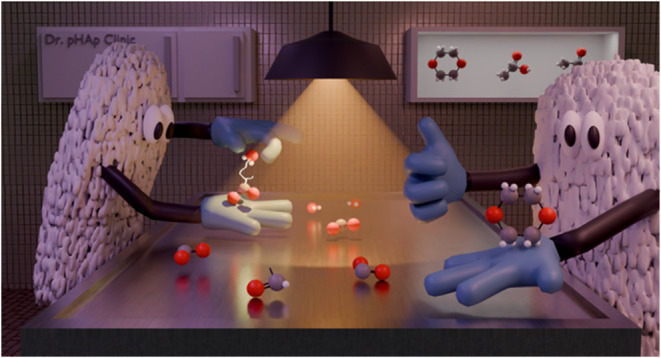

## Introduction

The use of CO_2_ as a feedstock reagent for the synthesis of valuable chemical products and industrial economy decarbonization is a hot topic.^1–5^ Currently, the vast majority of studies report the catalytic synthesis of small organic molecules, generally C_1_ or C_2_, and focus on yield enhancement. However, despite the energetic improvements made^6–9^ and the high additional value associated with CO_2_ reduction,^10^ the catalysts reported are still incapable of competing with industrial C_1–2_ synthesis processes.^11^ Such a trend drastically changes when focusing on longer organic molecules (C_3+_). Certainly, single-step CO_2_-to-C_3+_ fixation reactions are more interesting in terms of cost–benefit, as they immensely decrease the complexity of the aforementioned well-established industrial synthetic routes.^12^

Nonetheless, CO_2_-to-C_3+_ conversion reactions are challenging, as the growth and assembly of different species need to be promoted in front of stochastic thermodynamic fluctuations. Therefore, the strategic nanoscale material design is of utmost importance to develop next-generation industrial and sustainable catalysts. Among the novel design strategies, (S1) single/cluster atom catalysts, (S2) multiphasic layer systems, and (S3) vacancy engineering stand out. On the one hand, single/cluster atom catalysts offer a high degree of binding site customization, promoting C_3+_ production through synergetic material interactions. Accordingly, Kou et al. and Choukroun et al. reported MoN_2_ and Cu–Ag systems for the photo- and electrocatalytic CO_2_ conversion into hydrocarbons by careful binding site assembly and optimization.^13,14^ Despite their great potential, the main limitation of these systems is their high cost and complexity,^15–17^ limiting their scalability. Similarly, the coexistence of two materials in multiphasic layer systems (*i.e*., structural engineering of the catalysts’ interfaces) promotes the assembling of C–C bonds up to C_3+_ products, showing increased efficiency and selectivity.^18–20^ Within this framework, the design and tuning of heterojunction systems (*e.g*., *S-scheme and Z-scheme*) are also promising strategies for photocatalytic CO_2_ reactions.^20–22^ Lastly, vacancy engineering has emerged as an advanced design technique through lattice charge decompensation, promoting photon adsorption and boosting molecular adsorption.^23^ As reported in several studies, introducing oxygen^24–27^ or other element^28,29^ deficiencies enhances significantly the photocatalytic reaction yield for CO_2_ reduction reactions to CO and/or CH_4_. Nonetheless, as mentioned above, C_2_–C_3+_ production remains extremely difficult due to the complex chain growth mechanisms regulated by the adsorption/desorption balance.^30^ Therefore, studying such strategic-design combinations is a must in order to achieve more efficient pathways for value-added CO_2_-to-C_3+_ synthesis reactions, leading to the present study.

Permanently polarized hydroxyapatite (p-HAp) is a catalytically active and stable system based on a natural and abundant HAp ceramic. p-HAp is capable of fixing CO_2_ into mainly C_1_ and C_2_ products, such as formic acid, ethanol, or acetic acid, at mild conditions.^31–33^ The activation process, consisting of (1) ceramic calcination at 1000 °C, and (2) application of a thermally stimulated polarization (TSP) process (*i*.*e*., samples are exposed to 500 V at 1000 °C) has been reported to promote hydroxyl group vacancy generation and surface charge accumulation through electron delocalization, respectively, thus enabling carbon capture and conversion.^34,35^ It should also be highlighted that through a controlled hydroxyl-stabilized brushite (Bru) layer generation, achieved upon TSP conditions tuning, the catalyst selectivity is shifted toward C_2_ products.^36,37^

Although its use has never been explored for CO_2_-to-C_3+_ fixation reactions, p-HAp can be postulated as a great candidate because its design combines the S2 and S3 strategies. Furthermore, p-HAp suggests a multiple-binding site structure due to the possibility of fixing multiple acidic/basic reagents simultaneously,^38,39^ thus also fulfilling the objective of S1. Herein, the lack of previous experimental evidence reporting the synthesis of C_3+_ products has motivated a complete revision of the features of p-HAp and the reaction conditions reported. Particularly, the light interaction (*i.e*., density of states (DoS)) with the polarized catalyst and photocatalytic effects on carbon fixation have not been studied properly. Indeed, although p-HAp is currently described as a thermal catalyst, the customizable generation of vacancies (and the overall lattice refinement reported for its catalytic activation) suggests an unexplored self-photocatalytic potential. Accordingly, such a feature has been exploited through the synergistic effects resulting from the incorporation of nanoparticles such as TiO_2_ or ZrO_2_.^8,40^

In this work, the exploration and assessment of p-HAp-based systems, in which biphasic layer design and vacancy engineering strategies coexist, have been carried out using light irradiation. Accordingly, the catalytic activity of the prepared samples toward C_3+_ products has been tested under three irradiation conditions: (1) no light, (2) solar light, and (3) UV light at mild conditions, reaching the formation of up to C_4_ products. To elucidate the mechanisms behind this outstanding achievement, an exhaustive computational study supported by experimental characterization was carried out. More specifically, Density Functional Theory (DFT) calculations have been used to determine the DoS and perform band structure analyses, in addition to CO_2_ adsorption studies. Therefore, the roles of light irradiation and acidic/basic binding sites have been elucidated. Finally, the CO_2_ dissociation pathway has been studied computationally, confirming and further refining the experimental mechanisms proposed in the literature.^41^

## Results and Discussion

### Design and Characterization of the Catalysts

The HAp-based catalysts were prepared as porous scaffolds following a two-step process, as explained in the Materials and Methods section. Accordingly, the scaffolds were calcined at 1000 °C to remove the polymeric network (using a sacrificial template to generate pores) while generating OH^–^ vacancies in the crystal lattice, hereafter referred to as c-HAp. The generation of OH^–^ vacancies is aimed at increasing the density of the binding acidic/basic sites (see the discussion in the following sections) and the locally available electrical charges.^34^ The catalytic activation of the systems was completed using the TSP treatment. As extensively reported in the literature, such treatment leads to both a significant refinement of the HAp crystal lattice as well as to the permanent reorientation of the OH^–^ groups contained, which allows the delocalization of the generated electrical charges throughout the lattice, boosting its electrical properties.^34–37^

Besides, the TSP treatment can also be exploited to customize the HAp interface by producing hydroxyl-stabilized Bru as a coexisting phase. Previous works have suggested that Bru is able to increase the adsorption of ^•^CH_3_ radicals, tuning the selectivity toward C_2_ products.^36^ Inspired by such results and with the aim to obtain C_3+_ products, in this work, we go one step further by comparing a standard permanently polarized HAp catalyst (*i.e*., p-HAp, showing some minor Bru spots) with a biphasic system showing a large and homogeneous Bru interface (referred to as p-HAp/Bru). As seen in Figure 1a, p-HAp/Bru is obtained by increasing the temperature of the TSP treatment to 100 °C.

**Figure 1:**
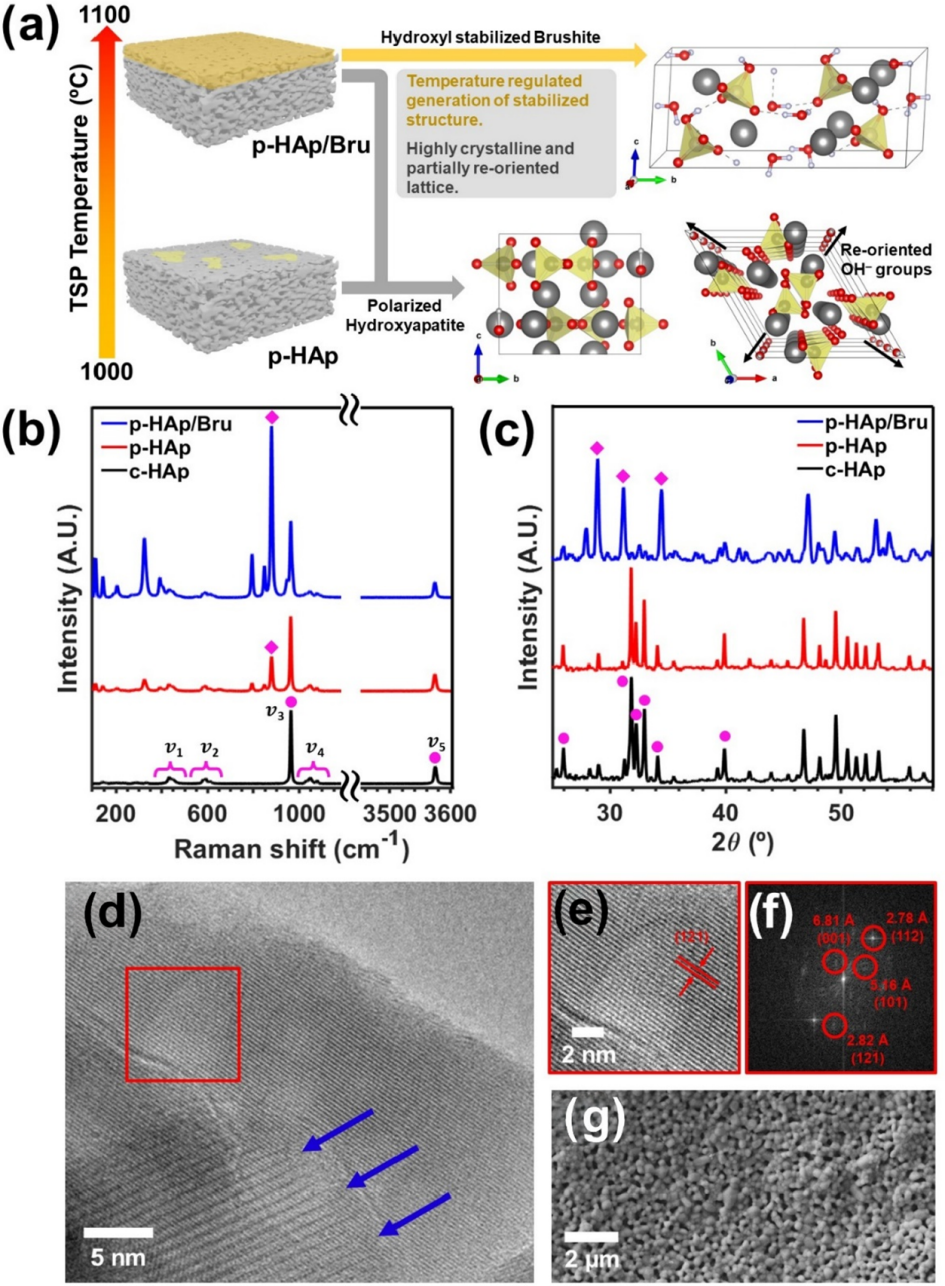
(a) Scheme with p-HAp and p-HAp/Bru porous catalyst crystal structures, where the biphasic outer layers can be observed. The role of hydroxyl groups in stabilizing Bru as a meta-phase during TSP treatment has been exhaustively discussed in the literature.^36^ (b) Surface area Raman spectra acquired for the three different systems with pink circles marking the characteristic HAp active modes υ_1_ = 400–480, υ_2_ = 570–625, υ_3_ = 965, υ_4_ = 1020–1095, and υ_5_ = 3574 cm^–1^, attributed to the double degenerate O–P–O bending mode, the triply degenerate asymmetric and symmetric P–O stretching modes, the triply degenerate O–P–O bending mode and stretching vibration of υ–OH peak at 3574 cm^–1^, respectively. A diamond shape is used to mark the 878 cm^–1^ Bru mode. (c) Representative WAXD diffractograms for the three samples, marked similarly to those described in (b). (d) HR-TEM image, where a highly crystalline p-HAp region can be observed. Fringe overlapping among differently oriented crystals, giving pseudopatterns, is marked with blue arrows. (e) Red inset from the former HR-TEM image, where the (121) crystallographic plane can be observed. (f) Fourier transform from the polycrystalline region with different planes. (g) SEM image of p-HAp, showing the sample pores.

To confirm proper sample preparation, Raman spectroscopy and wide-angle X-ray diffraction (WAXD) studies are presented in Figure 1b,c, respectively. As can be seen in Figure 1b, the HAp Raman active modes can be clearly observed in all samples (see complete peak assignment in the figure caption). Moreover, the presence of Bru (878 cm^–1^) in p-HAp and p-HAp/Bru confirms the proper HAp catalytic activation.^32^ It is worth noting that the area ratio between Bru and HAp (υ_1_) increases from ∼76% to ∼136%, confirming the presence of a larger and more homogeneous Bru interface for p-HAp/Bru (see also Raman surface data maps in Figure S1).

The Bru layer was further confirmed by WAXD, as shown in Figure 1c. While c-HAp and p-HAp present the characteristic HAp reflections corresponding to the (002), (211), (112), (300), (202), and (310) crystallographic plane reflections (2θ = 25.9°, 31.7°, 32.1°, 32.8°, 34.0°, and 39.8°, respectively; JCPDS card number 9-0077), the p-HAp/Bru diffractogram completely differs from the previous ones, displaying the characteristic (041), (2̅21) and (2̅02) crystallographic plane reflections attributed to Bru (2θ = 28.9°, 31.2°, and 34.5°, respectively; JCPDS card number 01-089-0377). Therefore, although its presence can be observed in both p-HAp and p-HAp/Bru, only the latter contains a relevant amount to be considered as a homogeneous layer.

Structural and morphological analyses carried out by high-resolution transmission electron microscopy (HR-TEM) (Figure 1d–f) and scanning electron microscopy (SEM) (Figure 1g) further confirmed the correct catalyst preparation. As seen in Figure 1d, the p-HAp samples display periodically aligned fringes associated with a high crystallinity degree, which is of utmost importance for ensuring proper material activation. Accordingly, the crystallographic planes (101), (121̅), and (112) with *d*-spacings of 5.16, 2.82, and 2.78 Å, respectively, can be observed in addition to the (001) polarization superstructure at 6.81 Å, which is characteristic of p-HAp systems.^32^ Although the p-HAp/Bru sample was also examined by HR-TEM, no Bru crystal lattice was observed, which was attributed to the HR-TEM sample preparation protocol (*e.g*., grinding of samples or suspension and sonication). Nonetheless, the HAp phase in the p-HAp/Bru samples displays the same fringe patterns as p-HAp (see Figure S2), confirming that the Bru layer generation does not affect the underlying polarization of HAp. Finally, morphological inspection of all samples proves that the synthesis process was carried out successfully, as highly porous scaffolds are obtained (Figure 1g), resulting in ∼16% porosity and an average pore diameter of 165 ± 73 nm.

### CO_2_ Fixation Reactions: From C_1_ to C_4_ Product Generation

As depicted in Figure 2a, the use of different irradiation sources has been considered as the second strategy to obtain C_3+_ products (*i.e*., the first approach was the generation of a consistent Bru interface). Therefore, CO_2_-fixation reactions have been performed in a custom-made continuous-flow reactor operating at 120 °C and under (1) no irradiation, (2) UV light irradiation, and (3) solar lamp irradiation conditions (all the details are found in the Materials and Methods section). To ensure reproducibility, all of the reactions have been carried out in triplicate. Accordingly, the error in the yields obtained was calculated by considering the standard deviation of all the repetitions. Although the quantification of the products adsorbed and desorbed has been performed by ^1^H NMR, the present work focuses on the products adsorbed in the catalysts since the study aims to break down, from a nanoscale point of view, the C_4_ product formation mechanism under mild and nonexpensive conditions rather than reporting CO_2_-fixation efficiencies and key performance indicators, which can still be found in Table S1. Nonetheless, the yields of the desorbed products are displayed in Figure S3, reaching the productions of 34.69 ± 1.52 and 0.11 ± 0.4 mmol·g_c_^–1^·h^–1^ for C_1–2_ and C_3–4_ products, respectively. Additionally, the stability and recyclability of the catalyst for the CO_2_ fixation reaction at temperatures lower than 600 °C have been demonstrated elsewhere.^33^ Hence, the feasibility and suitability of the process are demonstrated. Additionally, the blanks and controls of the reaction (*i.e*., a reaction without a catalyst and using c-HAp) are presented in Figure S4.

**Figure 2:**
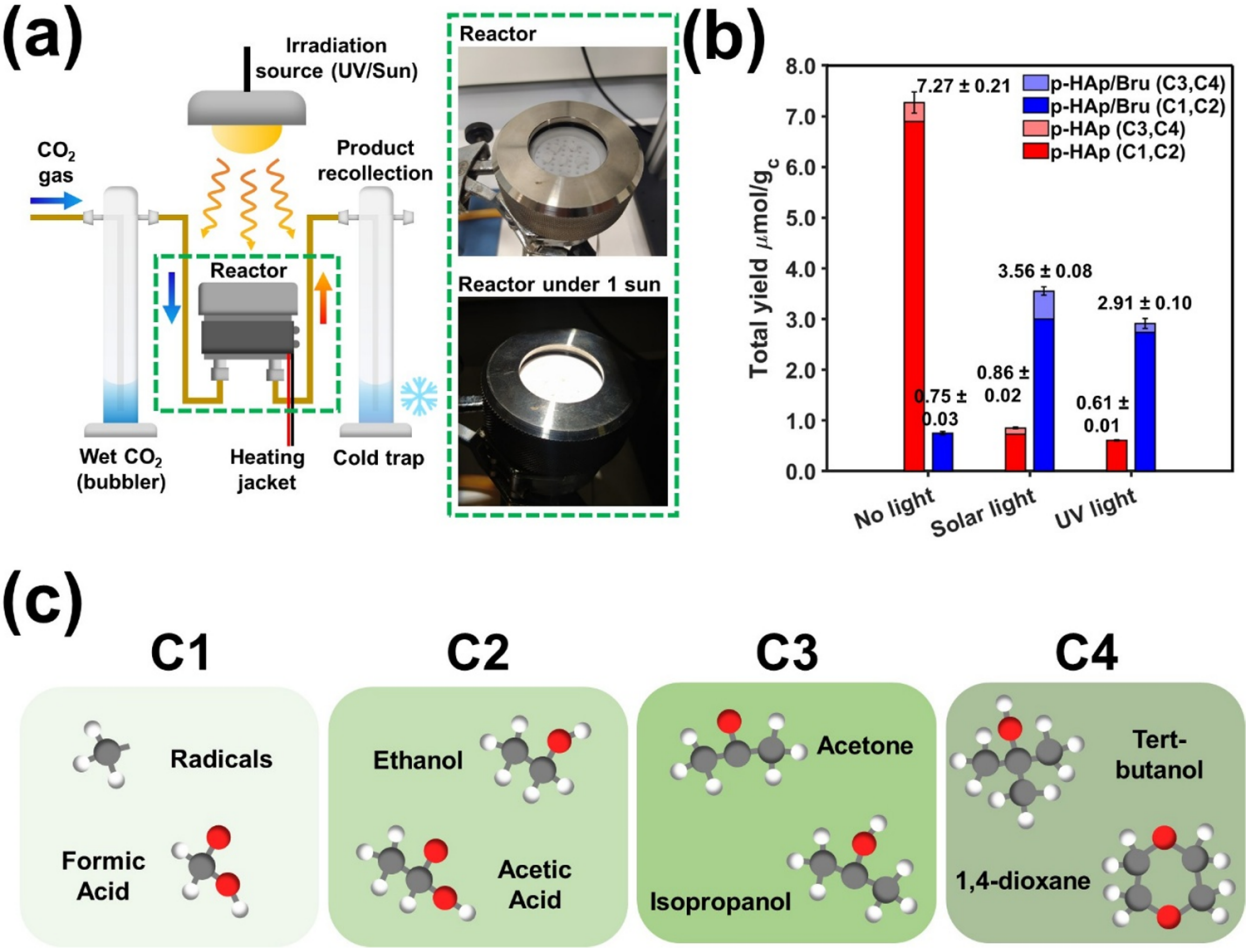
(a) Continuous-flow reactor setup scheme with real photo inset without/with solar light irradiation. (b) Adsorbed product yields for the p-HAp and p-HAp/Bru catalyst samples. A color gradient was used to identify the C_3–4_ portion among the total yield. (c) Different products were obtained from the photocatalytic reactions. The specific adsorbed product selectivity is displayed in Figure S5.

Accordingly, Figure 2b,c shows the yields and specific reaction products adsorbed on the catalyst and analyzed after 1 h of reaction. Besides, the specific adsorbed product selectivity and the most representative ^1^H NMR spectra are shown in Figures S5–S6, respectively. Interestingly, the C_4_ products, 1,4-dioxane and tert-butanol (presenting characteristic singlets at δ = 3.71 and 1.31 ppm, respectively), which have never been reported for catalytically active HAp, were detected in the solar and UV light reactions using both catalysts. In addition to being C4 products with high added value, it is worth highlighting the fact that a cyclic compound is synthesized using only a single-step CO_2_-fixation reaction. The chemical shifts (δ) associated with the other products are listed in Table S2.

As can be seen in Figure 2b, the presence of the Bru interphase and light irradiation presents a strong synergy toward the production of C_3–4_ products, suggesting a stronger adsorption capacity (supported also by the trend in the desorbed products of Figure S3). More specifically, the maximum yield of p-HAp is achieved without light irradiation (*i*.*e*., 7.27 ± 0.21 μmol·g_c_^–1^ of C_1–2_ products; this value is reduced up to 88% and 91% for solar and UV light irradiation, respectively), p-HAp/Bru increases its production from 0.75 ± 0.03 μmol·g_c_^–1^ (no irradiation) to 3.56 ± 0.08 and 2.91 ± 0.10 μmol/g_c_ of C_3–4_ products under solar and UV light irradiation, respectively. Note also that in terms of CO_2_ fixation, both yields are almost equivalent. More exhaustive analyses revealed that the selectivity toward C_3–4_ products (see Table 1 and Figure S5) shifted depending on the irradiation conditions. Overall, the results suggest the presence of binding sites with equivalent catalytic activity but customizable selectivity, the paradigm of the so-called *catalytic plasticity*.^36^

**Table 1:** Adsorbed (Catalyst) and Desorbed (Cold Trap) C_1_ + C_2_ and C_3_ + C_4_ Product Selectivities Obtained for the Continuous-Flow Reactions for p-HAp and p-HAp/Bru Samples under Different Light Irradiation Conditions

adsorbed products			
		selectivity %	
catalyst	irradiation	C_1_ + C_2_	C_3_ + C_4_
p-HAp	no	94.94 ± 3.04	5.06 ± 0.08
	solar light	85.33 ± 4.01	14.67 ± 1.07
	UV light	100.00 ± 0.00	0.00 ± 0.00
p-HAp/Bru	no	100.00 ± 0.00	0.00 ± 0.00
	solar light	84.42 ± 2.78	15.58 ± 0.54
	UV light	93.93 ± 4.22	6.07 ± 0.26

desorbed products			
		selectivity %	
catalyst	irradiation	C_1_ + C_2_	C_3_ + C_4_
p-HAp	no	100.00 ± 0.00	0.00 ± 0.00
	solar light	90.66 ± 3.39	9.34 ± 0.24
	UV light	98.65 ± 5.84	1.35 ± 0.07
p-HAp/Bru	no	93.99 ± 2.62	6.01 ± 1.78
	solar light	53.46 ± 3.40	46.54 ± 1.43
	UV light	97.27 ± 5.97	2.73 ± 0.03

### Breaking Down the HAp-Based Catalysts Used for the Production of C_3+_ Products

Arnau et al. proposed the CO_2_-to-C_1–2_ product formation mechanism catalyzed by p-HAp without light irradiation, which was elucidated using near-ambient pressure X-ray photoelectron spectroscopy (NAP-XPS) and demonstrated by isotope-labeling ^13^CO_2_ experiments.^41^ In this sense, p-HAp was considered a thermal catalyst with uniquely enhanced electronic properties. However, the recent possibility to produce C_3–4_ products upon the application of light has motivated a complete revision of the system, focusing specifically on understanding the role of light (*i.e*., the photocatalytic contribution) and its interaction with HAp-based catalysts. In addition, the shift from C_1–2_ (which might be produced due to random thermodynamic fluctuations of the anchored species) to C_3–4_ products also suggests that HAp-based catalysts possess a unique set of different binding sites that have not been studied until now. Accordingly, this section sheds some light on this through a mixed experimental and computational study.

#### Electron Excitation and Conduction

Density functional theory (DFT) calculations, aimed to determine the density of states (DoS) and band diagrams (BD), were performed for HAp, c-HAp, p-HAp, and Bru. As preliminary work, the most appropriate computational methods were explored. Accordingly, the band gap (B_g_) has been calculated considering different assumptions (Figures S7 and S8) and finally compared to the experimental B_g_ through Tauc plot analysis (Figure S9), which has been built from the UV–vis absorption spectra presented in Figure 3. Herein, to incorporate the structural lattice modifications caused by the catalytic activation (*i.e*., vacancy generation and OH^–^ reorientation), 1 × 1 x 2 supercells were considered for all systems. Details of the exact computational methods used hereafter are provided in the Materials and Methods section. The resulting DoS is presented in Figure 3a. As can be seen, the 50% hydroxyl vacancies (experimentally determined in the literature)^34^ result in two trap states appearing in the middle of the HAp band gap region. Each trap state was attributed to the removal of a single hydroxyl group within the original supercell. Such a result has been further corroborated in Figure S10a by considering only 25% hydroxyl group vacancies (c25-HAp). After TSP application, both states merge into a single trap state (see the green inset in Figure 3a) without losing state availability as the total areas are maintained (Figure S10b). Certainly, the generation of trap states along the band gap is of great advantage for photocatalytic materials because the energetic requirements to excite an electron from the valence band (VB) to a more energetic state (trap state, instead of the conduction band (CB)) are lowered.^42–44^ Accordingly, the customization of the band gap can be clearly seen in Figure 3b. Interestingly, all of the structural modifications result in a decrease of both the band gap and the trap state (which are found within the range of 42–58% of the CB electron excitation energy) as compared to pristine HAp. Thus, all samples exhibit absorption in both the UV and solar light domains after the applied treatments. However, note the abrupt change in the trend observed for p-HAp. Such an increment might arise from steric interferences, further confirming the stabilization of the polarized structure (*i.e*., the permanent state is reached).^35^ More specifically, this stabilization is experimentally supported by the presence of a superstructure (Figure 1f) and computationally by the changes in the crystallographic parameters of the relaxed p-HAp supercell (Figure S11). Additionally, the UV–vis spectra presented in Figure 3c,d experimentally confirm the simulation results obtained. As can be seen in Figure 3c, p-HAp exhibits three main absorption domains at 220 nm (A), 277 nm (B), and 363 nm (C), which have been attributed, according to the band gaps obtained in Figure 3b, to the Bru phase (A) and the combined p-HAp features of the CB (B) and trap states (C). The p-HAp/Bru UV–vis spectrum in Figure 3d further confirms the Bru assignment (*i.e*., see the area increase of 1.7% in A and decrease of 10.5% in B), and the UV–vis spectra of pristine HAp (Figure S12) confirm the B and C assignments.

**Figure 3:**
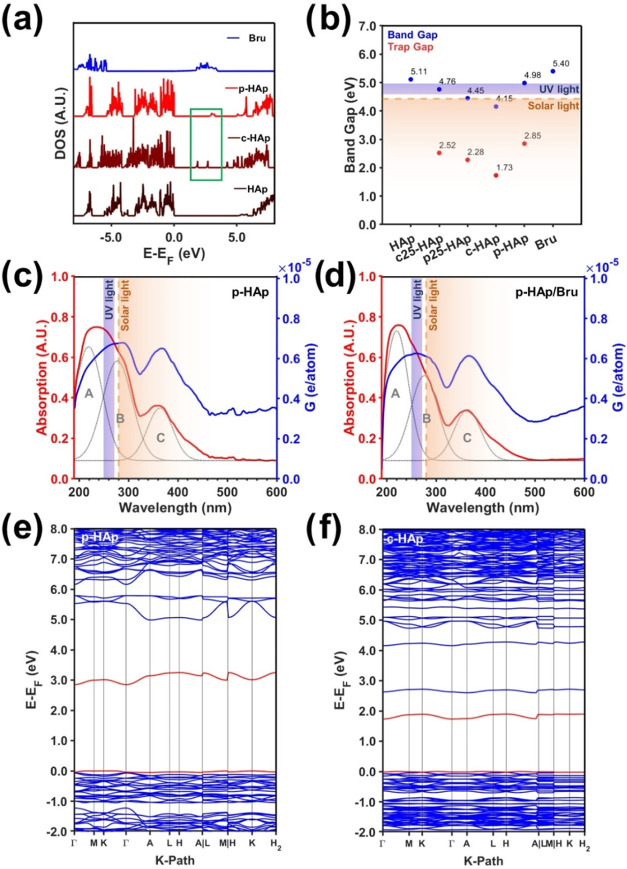
(a) DoS calculations for the different structures modeled. The green inset shows the p-HAp and c-HAp band gap regions, where trap states appear. (b) Comparison between band gaps and trap states decreased band gap. The solar and UV light wavelength regions are displayed in orange and blue color gradients, respectively, to illustrate photoelectron excitation. (c and d) UV–vis spectra of the p-HAp and p-HAp/Bru catalyst samples (red) and their corresponding charge carrier generations (blue) with a graphic representation of light irradiation domains. (e and f) Band diagrams of the p-HAp and c-HAp samples, respectively. Red is used to identify the last valence band and the first trap state band.

Further characterization through BD calculations (Figures 3e,f and S13) determined that the electron transitions to trap states have an indirect minimum energy (Table S3). Nonetheless, direct transitions only differ by <0.05 eV; thus, both transitions can be assumed to be due to the contribution of lattice vibrations (phonons) at 120 °C (conditions of the catalytic tests). Overall, the polarization treatment not only reduces the band gap energy of p-HAp with respect to HAp but also generates trap states that enable sunlight and UV light to promote electrons toward higher energy states, which could be used for both C=O bond cleavage and enhancement of the reaction kinetics of the intermediate species. In addition, this result is experimentally supported in Figure 3c,d, where a photocarrier generation within 0.5–0.6·10^–5^ e/atom has been calculated, proving remarkable light absorbing capabilities.

On the other hand, understanding electron conduction is crucial for assessing the migration of excited electrons toward the binding sites, avoiding recombination phenomena. To do so, electron impedance spectroscopy (EIS) measurements were carried out for c-HAp, p-HAp, and p-HAp/Bru, as shown in Figure 4. The Nyquist plot presented in Figure 4a confirms that the conductivity of p-HAp is enhanced compared to that of c-HAp. However, p-HAp/Bru shows the opposite behavior (*i.e*., a larger semicircle: higher charge accumulation), which is attributed to the Bru layer generated on top of the sample. The equivalent circuits used to analyze the EIS data presented in Figure 4b have been previously validated in the literature.^33^ Thus, the aforementioned behavior is confirmed when inspecting the circuit parameters in Figure 4c,d, which reveal that, although the polarized HAp bulk resistance (R_b_) and capacitance (C_b_) are not significantly different between the p-HAp and p-HAp/Bru samples, the Bru layer resistance (R_γ_) and capacitance (modeled as a constant phase element (CPE_γ_)) differ by 1 order of magnitude.

**Figure 4:**
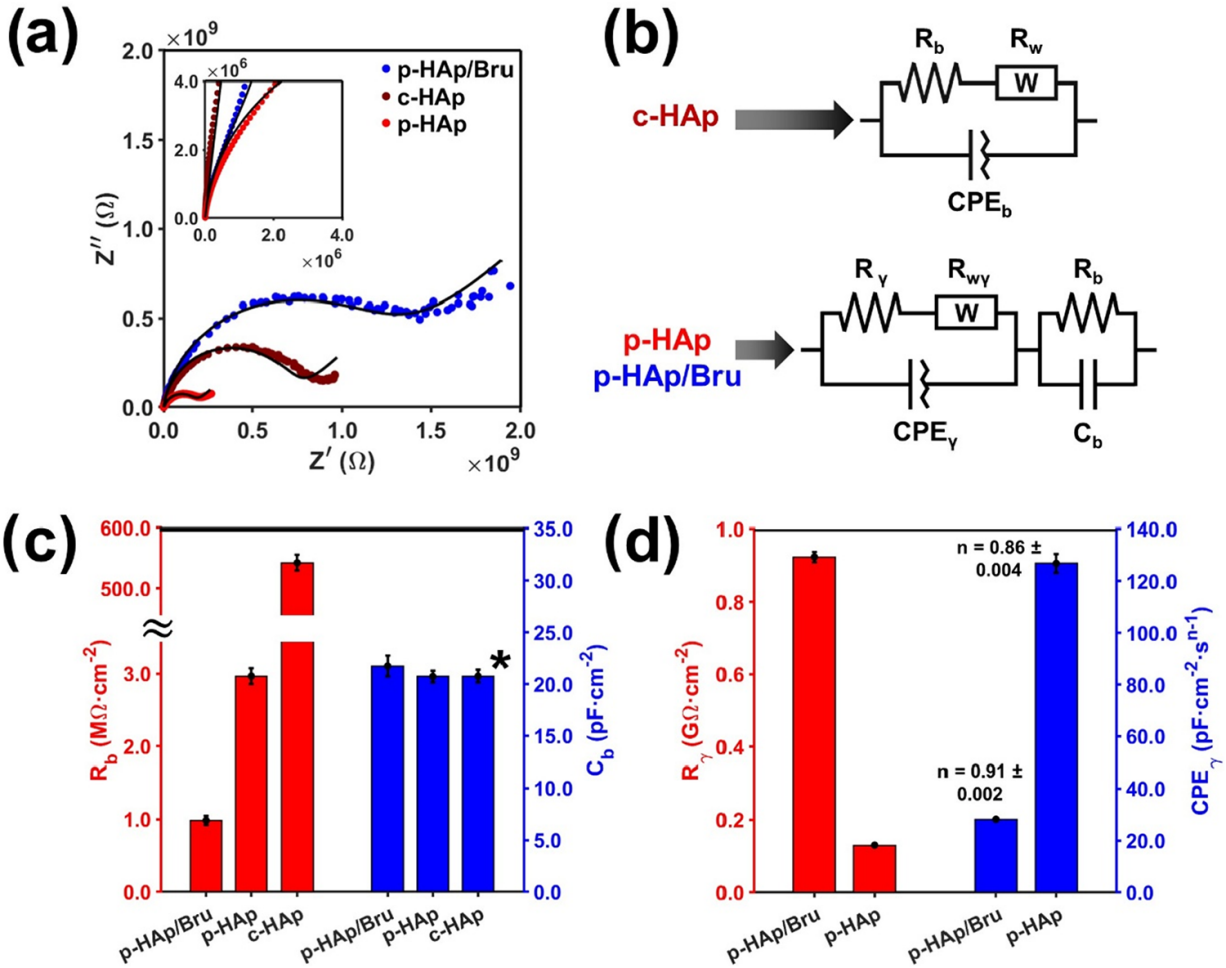
(a) Nyquist plots for the two catalytically active samples and the control. The inset shows that p-HAp and p-HAp/Bru behave very similarly at high frequencies but differ at lower frequencies, thus displaying the high capacitive behavior of p-HAp/Bru. (b) Equivalent circuits for c-HAp, p-HAp, and p-HAp/Bru. (c) Sample bulk resistance (red) and capacitance (blue). c-HAp capacitance is marked with a star since the equivalent circuit model contains a constant phase element of *n* = ∼0.93, which can be considered an almost perfect capacitor. All parameters can be found in Table S4. (d) Surface Bru resistance and capacitance contribution to p-HAp and p-HAp/Bru samples.

Hence, while p-HAp favors the fast migration of the generated photocarriers, Bru exhibits a capacitive behavior gathering locations where electrons tend to accumulate, granting their availability to the surrounding binding sites. As seen in both the adsorbed and desorbed products (Figures 2b and S3, respectively), such synergy promotes the growth of C_3+_ molecules in front of the characteristic fast desorption of C_1–2_ seen for p-HAp without irradiation. Moreover, the role of Bru (*i.e*., charge accumulation and, thus, higher adsorption rate of the intermediates) is further confirmed experimentally in Figure S3 as the light-excited electron of p-HAp tends to present a higher desorption rate. Nonetheless, a proper HAp/Bru balance must be achieved, as a complete and dense surface insulating layer (*i*.*e*., the higher Bru coverage in p-HAp/Bru reached ∼1 GΩ/cm^2^) avoids electron transfer to the binding sites, lowering the catalytic yields. As shown in Figure 2b, such a point is extremely relevant for nonirradiated reactions. However, as seen in Figures 1b and S1, a portion of p-HAp is preserved on the surface of p-HAp/Bru, thus ensuring that the catalytic activity of the system is not deactivated. In this sense, the yield enhancement phenomena observed under light irradiation (Figure 2b) are explained through the additional photoelectron excitation generation at the HAp binding site, enabling to partially overcome the isolating Bru hindrance through its electrical saturation.

#### Binding Site Theoretical Studies

Although the catalytic activity of p-HAp and p-HAp/Bru can be explained from light-excitation and electrical perspectives, a deeper study of the binding sites is required to understand the selectivity shifts between C_1–2_ and C_3–4_. As mentioned above, the possibility of producing C_3–4_ suggests the existence of multiple binding sites in p-HAp that are close enough to promote the interaction between different species over random thermodynamic fluctuations. To do so, the CO_2_ adsorption energies for the main HR-TEM crystallographic planes (001), (101), and (121) were calculated using DFT, considering 14 dispositions (referred to as P#) among all possible binding sites. More specifically for the acid sites: Ca^2+^ I (distorted octahedron with a hydroxyl group), Ca^2+^ II (metaprism built from six PO_4_^3–^ tetrahedra), crystallographically nonequivalent sites, P^5+^ (PO_4_^3–^ tetrahedra) and OH^–^ vacancy; and the basic sites, O I (bonded to Ca I) and O II (close neighbors to Ca II).^45^ The specific crystallographic positions of each binding site are shown in Scheme S1.

The calculated energies are depicted as a heatmap in Figure 5a, while Figure 5b–d display the C–O bond length change (%), the CO_2_ dihedral angle change (%), and the adsorption positions for the (001) plane, respectively. The (001) plane is chosen to be displayed in the main text due to its slab simplicity and comprehensibility compared to the (101) and (121) planes, which are presented in Figures S14 and S15. As proposed, both (001) and (101) planes reveal the existence of multiple abundant binding sites with similar adsorption energies close to −1 eV. As can be seen, although the P1 binding site for the (001) plane appears to be the most suitable candidate with a value of −7.37 eV, this might be overestimated. That is, critically observing the slab corresponding to the (001) plane, it can be seen that the Ca I surface atoms are 3-fold oxygen-coordinated instead of 6-fold, becoming highly charged centers that attract CO_2_ molecules. Indeed, it can be assumed that passivation would partially occur under experimental conditions, deactivating them. Therefore, the most favorable binding site is P10 for the (101) plane, achieving −3.57 eV, where the C atoms from CO_2_ molecules are adsorbed to a close vacancy O I (see Figure S14c). Interestingly, P12 (vacancy site) and P13 (Ca II:O I) for the (101) plane binding sites present a similar disposition but with slightly lower adsorption energy (∼0.7 eV). This result indicates that the vacancy steric effects play a major role in enhancing the adsorption power of the binding sites. Indeed, the calculations performed confirm the hypotheses proposed in the literature, showing that the homogeneous distribution of vacancies among the HAp bulk and charge delocalization through hydroxyl reorientation favor the formation of localized surface charge imbalances, boosting the CO_2_ attraction onto multiple specific sites. Finally, it is worth mentioning that some of the high positive adsorption values (*i.e*., P12 (001): +6.85 eV and P4 (121): +3.38 eV) can only be obtained by applying bond constraints, thus imposing the energetic relaxation into the desired site (see the Materials and Methods section). Therefore, rather than considering the absolute value, they must be understood as “virtually” energetically unstable sites.

**Figure 5:**
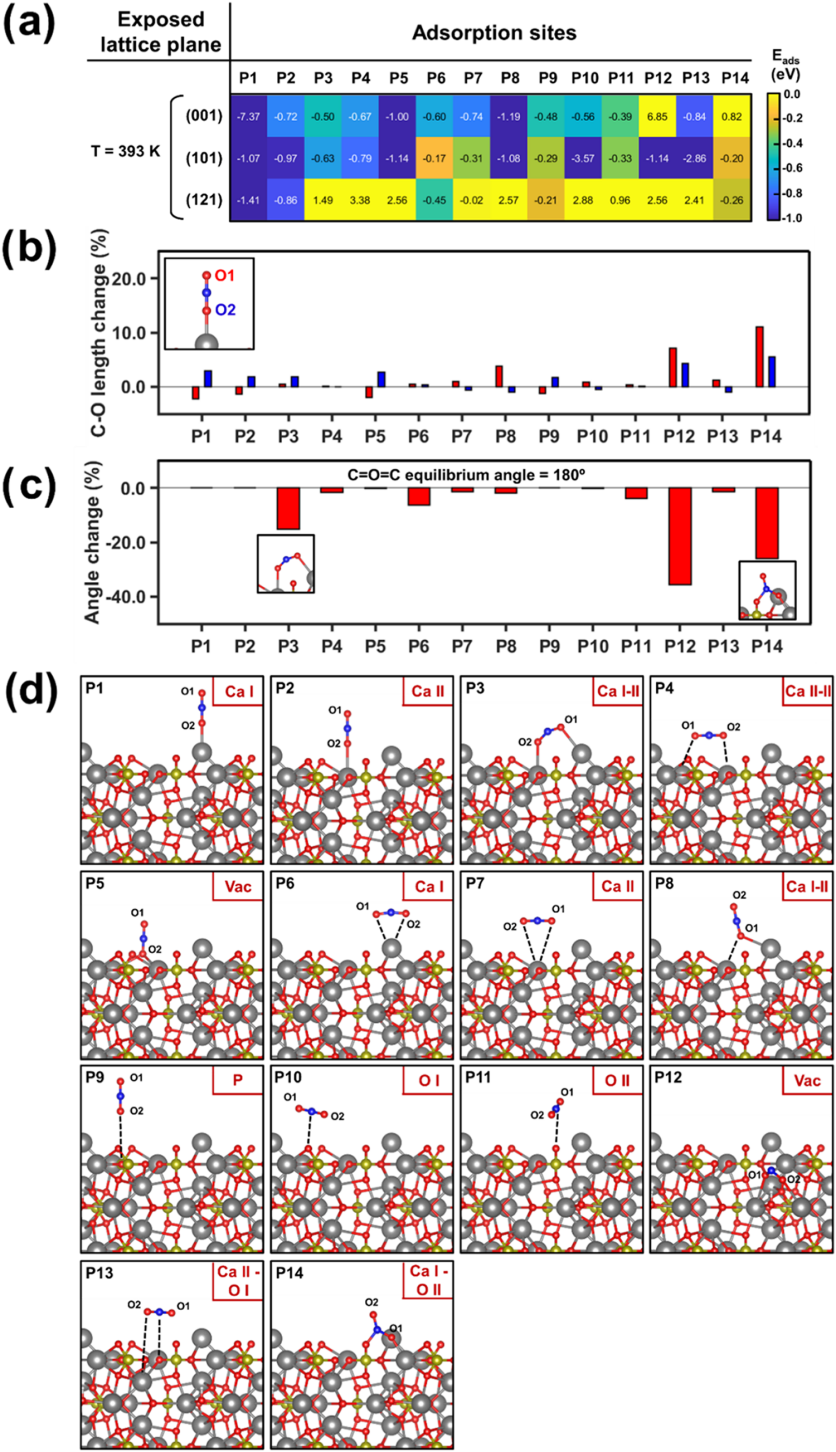
(a) Adsorption energy heatmaps for the (001), (101), and (121) crystallographic planes of p-HAp. All energies were calculated using the equation in the Materials and Methods section at a 120 °C simulating reaction temperature. (b) Bar chart displaying the C–O bond length displacement % with respect to the original equilibrium value. Red and blue colors were used to identify each of the two oxygen atoms composing the CO_2_ molecules. (c) CO_2_ molecular bond angle change % with respect to the equilibrium. (d) 14 binding sites/positions studied, where an adsorbed CO_2_ molecule has relaxed to the (001) crystallographic plane.

On the other hand, it is also worth noting that the C–O bond length (Figure 5b) and angle (Figure 5c) are also influenced by the adsorption energy according to the following scenarios: (1) a single CO_2_ oxygen coordinated with the binding site as in P1, P2, or P5; and (2) two atoms from the CO_2_ molecule are coordinated with one or more binding sites, for instance, P3 or P4. For the first case, a single anchor point promotes quasi-equilibrium angle stability, while <5% bond stretching achieves negative adsorption energies in all cases. However, in the second case, adsorbed molecules are subjected to two binding sites, which leads to significant angle deviation (>10%) from the equilibrium case and induces notable bond stretching. These geometric perturbations result in either a nonfavorable positive adsorption energy for the binding site, atom distance >2 Å, or a high negative energy for closely packed molecules to the surface, which find steric charge concurrence, as in P10 and P13 for the (101) plane.

Overall, the heterogeneous availability of multiple CO_2_ adsorption sites on p-HAp further supports the C_3+_ growth mechanism proposed above. Indeed, the study of the binding sites highlights the unique structural properties of p-HAp, which are electronically promoted through light irradiation and the incorporation of Bru as a biphasic interface. In this sense, the fact that both P10 and P13 are located in the (101) plane suggests that their exposure is of utmost importance. Accordingly, we are currently studying how to promote its growth during sample preparation, aiming to break through the 15% C_3–4_ selectivity threshold obtained in the present work.

#### C_3_–C_4_ Assembly: Cleaving the C–O Bond

A further step toward understanding the C_3_–C_4_ assembly was taken by studying the charge density accumulation/depletion when simultaneously placing five CO_2_ molecules onto the (001), (101), and (121) crystallographic planes (see Figures 6a,b and S16, S17; respectively). As expected, oxygen atoms exhibit charge accumulation, which would lead to H^+^ (*i.e*., from water) attraction and OH^–^ generation, leaving lattice-bonded carbonyl groups. When dispersing five CO_2_ molecules on the (101) slab, 4/5 relaxed onto adsorption P1 sites (6-fold oxygen-coordinated calcium atoms), while the remaining one occupied the P10 position. Figures 6a,b focus on the P10 region, showing electronic charge density polarization since the surrounding calcium and oxygen atoms from the lattice attract CO_2_, inducing C–O bond stress. In fact, Bader charge analysis (Figures 6c,e) shows that the P10 molecular charge balance is affected by +1.33e^–^ with respect to equilibrium (Figure 6c). That is, the P10 molecule charge experiences a massive electronic pull compared to other adsorbed molecules, attributed to the vicinity of the vacancy. This result suggests that p-HAp vacancies could act as oxygen attraction centers, breaking the C–O bond.

**Figure 6:**
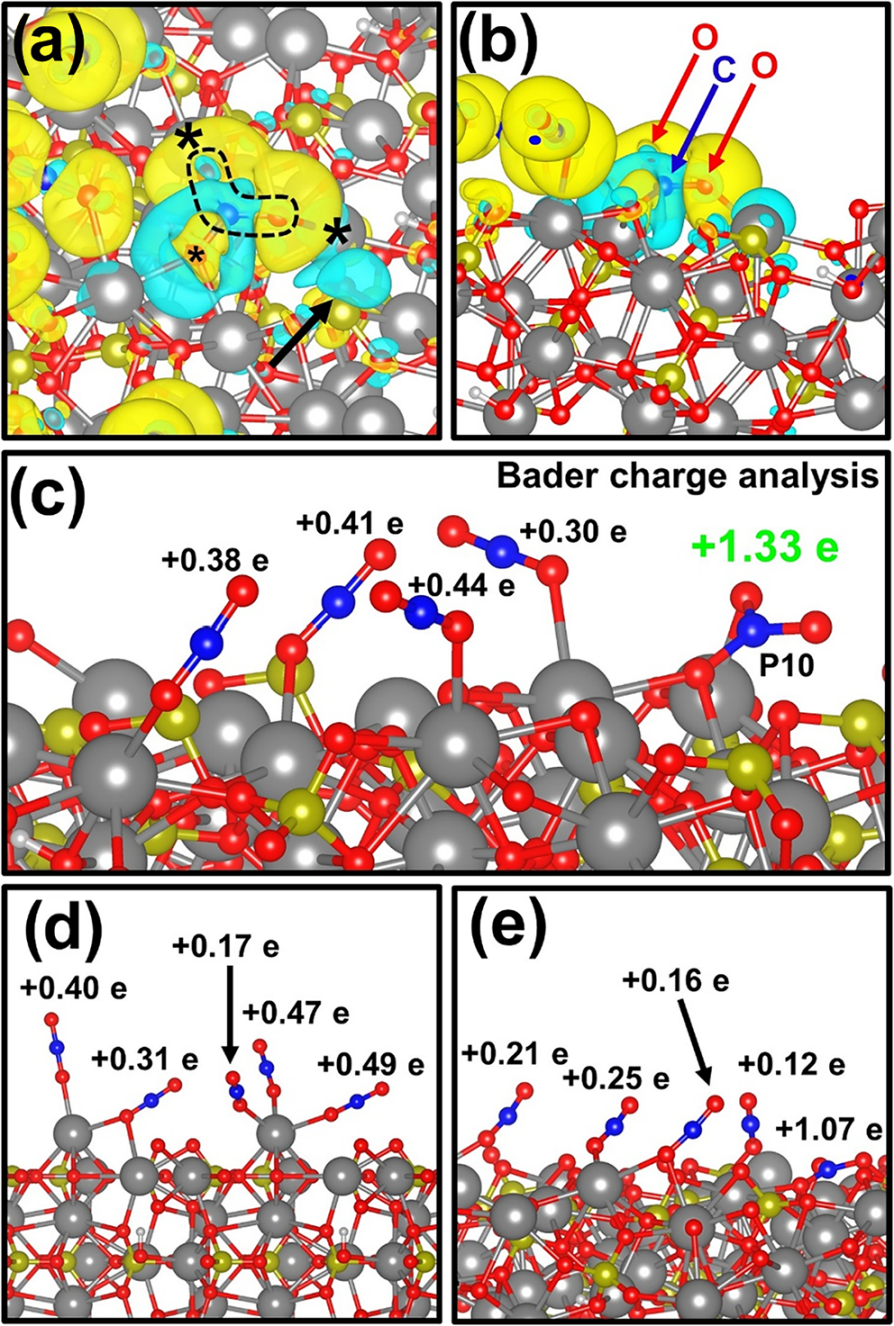
Five CO_2_ molecules simultaneously relax on the (101) p-HAp surface. (a) Charge accumulation/depletion (yellow/blue) for the molecule adsorbed at the P10 site. Stars have been used to mark the binding of calcium and oxygen to the oxygen and carbon atoms of the molecule, respectively. (b) Different angles for the same adsorbed molecule in the P10 position. (c) Bader charges for the 5 adsorbed molecules at the (101) slab. The positive charge sign indicates that molecules are adsorbed at the acidic sites of the p-HAp surface. (d, e) Bader charges for the (001) and (121) slabs, respectively.

Interestingly, the analysis of the P1 sites reveals the presence of multiple CO_2_ molecules bonded to the same site but with lower Bader charges. Consistently, the same behavior is also observed for the (001) and (121) planes (see highlights in Figure 6d,e). Such a feature is crucial to demonstrate the capabilities of p-HAp to produce C_3+_ products according to the mechanisms proposed above. Indeed, P1 sites can promote the dimerization of intermediates with higher adsorption/desorption kinetics. The higher electronic pull of P10 can attract such intermediates acting as the root of C_3+_ growth (*i.e.*, note its higher binding energy, as shown in Figure 5a). To further study this interaction, Figure 7a depicts a wider image of the charge accumulation/depletion of the 5 molecules relaxed on top of the (101) plane for p-HAp. As can be seen, having an adsorbed molecule at the P10 position does not prevent other CO_2_ molecules from adsorbing at close range, with the C–C distance between the P10 molecule and the first closest neighbor being 4.85 Å, while those of the second and third closest neighbors are 6.64 and 8.06 Å, respectively (see dashed lines in Figure 7a).

**Figure 7:**
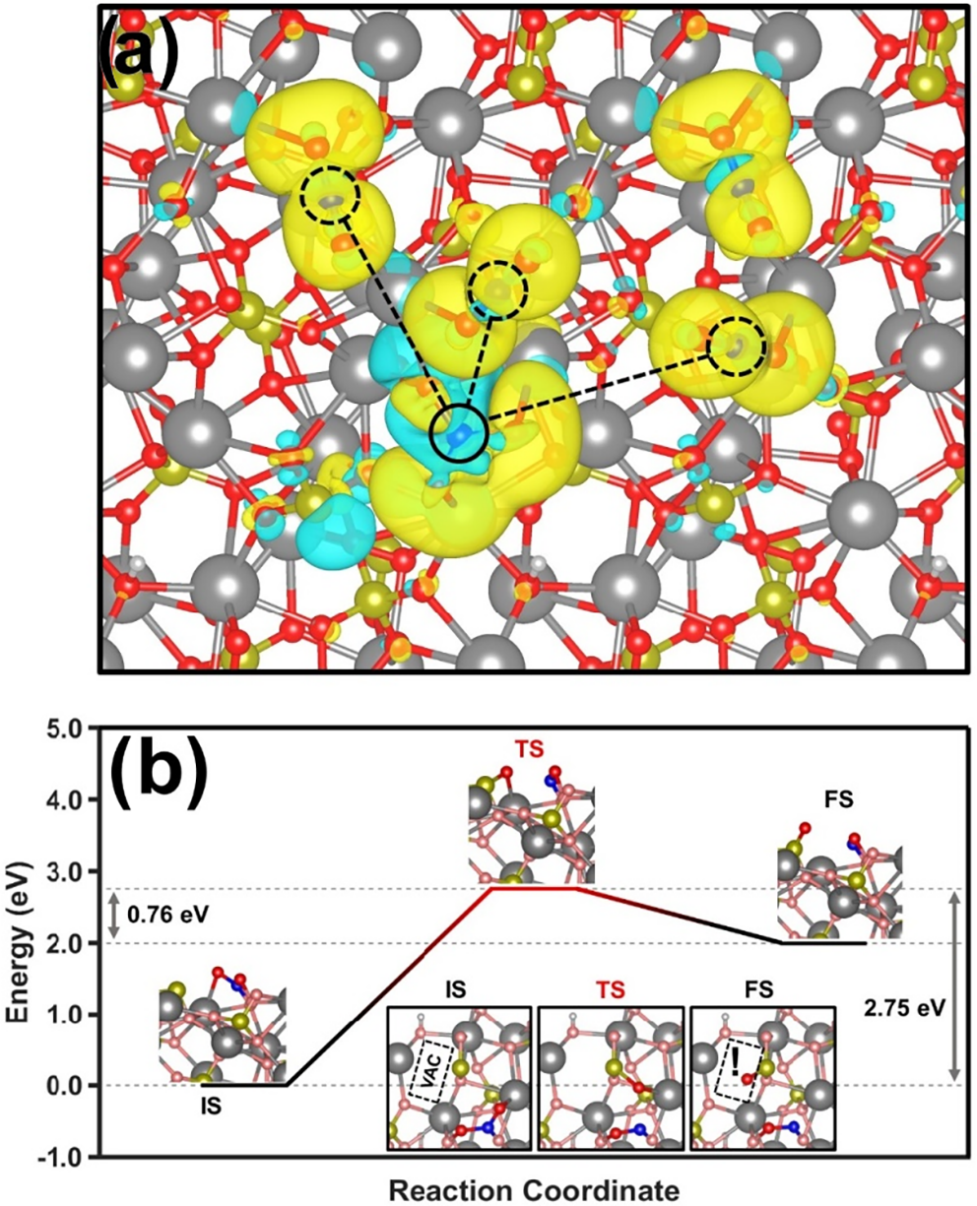
(a) Relaxed DFT geometry for five CO_2_ molecules on top of the (101) plane of p-HAp. Dashed lines and circumferences mark the vicinity neighbors to the P10 (straight line) highest-energy binding site. Charge accumulation/depletion is identified by yellow/blue, respectively. (b) Dissociation pathway for the CO_2_ molecules calculated using NEB. The initial state (IS), transition state (TS), and final state (FS) can be observed.

Finally, the Nudge Elastic Band (NEB) calculation was performed to determine the C–O bond cleavage, with a P10 adsorbed molecule as the initial state and the relaxed oxygen and carbonyl groups around the P10 location as the final state. The obtained reaction coordinate is displayed in Figure 7b, where a 2.75 eV energetic barrier and a more favorable final energetic state (as compared to the transition state) are achieved. Despite the energetic feasibility observed, overcoming the energetic barrier obtained needs further exploration. This is because, although the photocatalytic contribution of the system might be used to surpass such a barrier, from an experimental point of view, the presence or absence of light does not hinder the production of carbon products since CO_2_ bond cleavage occurs in both processes (see Figures 2 and S3). We attribute such behavior to the uniquely enhanced electron availability and surficial accumulation of the permanently polarized systems studied. Certainly, their role in no-light reaction conditions must be studied thoroughly. In addition, in the insets of Figure 7b, it can be observed that although the position of the carbonyl group is not affected, the adsorbed bond-free oxygen relaxes onto an energy minimum found at the vacancy, stressing its role as an adsorption enhancer. Additionally, it is worth mentioning that such a location is ideal to produce OH^–^ radicals due to the enhanced proton conductivity of p-HAp. That is, the vacancies are located in the realigned OH^–^ columns of p-HAp with enhanced proton conductivity. Overall, the presence of multiple binding sites with different adsorption energies, and thus, different reaction times, promotes the reaction of multiple intermediates derived from the presence of a partially dissociated C≡O species (experimentally observed in prior studies)^41^ and H–C–H species (supported by the detection of radical groups in the ^1^H NMR spectra in Figure S6) anchored in the catalyst. Interestingly, while the dimerization and trimerization of the carbidic species result in the formation of ethanol and acetone, respectively,^31^ tert-butanol can be obtained through the nucleophilic attack of the anchored H–C–H species on the central carbon of acetone (*i*.*e*., resembling the known reaction process with a Grignard reagent). In this sense, the complete reaction mechanism of 1,4-dioxane appears to be more complex and requires further experimental evidence for the coupling of different intermediates of the reaction.

## Conclusions

The present work explores the use of p-HAp and p-HAp/Bru biphasic systems as photothermal catalysts, reporting the production of C_3–4_ products with 15% selectivity. Successful reactions have been carried out under mild conditions (*i.e*., 120 °C and solar light irradiation), which is the first time that this achievement has been reported.

The mechanism behind C_3–4_ growth has been elucidated through systematic experimental and computational characterization studies. Hence, hydroxyl group vacancy engineering has been proven to be a key strategy to tune the photocatalytic activity of HAp-based catalysts. More specifically, the OH^–^ vacancy generation is directly related to the formation of trap states in the HAp band gap, thus offering the photoexcited electron generation at higher light wavelengths. Such photoexcited electrons can delocalize throughout the p-HAp bulk and migrate to the surface of the catalyst due to the TSP treatment. Besides, Bru clusters at the interface promote surface charge accumulation. Therefore, both features lead to remarkable synergy as not only are the generated intermediates strongly adsorbed on the p-HAp surface, but their reactivity is also higher. Nonetheless, a proper p-HAp/Bru balance at the interphase must be achieved, as large amounts of Bru reduce the electrical conductivity of p-HAp, hindering the catalytic efficiency.

The DTF calculations reveal a set of multiple binding sites in p-HAp with different adsorption energies. Accordingly, the analysis of several CO_2_ molecules simultaneously relaxed at the (101) crystallographic plane (among other p-HAp slabs considered) confirms the presence of a strong binding center (P10: O I, 3.57 eV) that acts as a root for the growth. Furthermore, such a binding site is surrounded by Ca^2+^ binding sites capable of attracting multiple CO_2_ species, promoting their dimerization and subsequent migration to the P10 site due to their close proximity. Critically thinking, although the selectivity is still low, it must be taken into account that both materials studied are highly abundant and cheap ceramics (compared to conventional metallic catalysts) and that very mild conditions are required for the reactions, allowing a fast, inexpensive, and environmentally friendly scalability of the technology. Moreover, the mechanistic insights reported suggest that further optimization (such as exposing the (101) crystallographic plane) can be done to improve the selectivity toward C_3–4_ products. Overall, the present work sets a precedent as it is the first time that an abundant and low-cost catalyst is capable of converting CO_2_ into C_4_ products at mild conditions. Although several efforts have been devoted to controlling the selectivity of p-HAp systems in previous studies through lattice modifications or the incorporation of TiO_2_ nanoparticles, only C1–C2 products were obtained (continuous reaction conditions), or some residual acetone (C3; selectivity <2%; batch reaction conditions) was obtained. Herein, the control on the brushite interphase and UV illumination allowed the production of C3–C4 products with a selectivity considerably higher under continuous reaction conditions, thus offering a promising industrial solution to minimize the effects of this greenhouse gas while promoting a circular economy model.

## Materials and Methods

### Hap-Based Catalyst Preparation

Synthetic hydroxyapatite (HAp) was produced through hydrothermal synthesis by mixing (NH_4_)_2_HPO_4_ (CAS: 7783-28-0) and Ca­(NO_3_)_2_ (CAS: 13477-34-4) solutions in basic media (pH 11) according to the 1.66 Ca/P HAp stoichiometric ratio. After 1 h of aging, a hydrothermal process (150 °C, 24 h) using autoclaves Digestec DAB-2 was applied to the mixtures, resulting in a precipitate powder extracted by centrifugation (two water washes followed by two additional 60/40 v/v ethanol/water washes) and lyophilization. Ultraporous HAp rectangular scaffolds (∼0.5 cm × 0.5 cm × 0.2 cm) were prepared using a Pluronic F-127 hydrogel-mediated process and later calcined, as reported elsewhere,^46^ obtaining the blank controls named c-HAp (calcined HAp). Finally, the TSP process was applied to c-HAp samples, converting them into p-HAp (polarized HAp) and p-HAp/Bru (hydroxyl-stabilized Brushite + p-HAp) by applying 500 V at 100 °C and 500 V at 1100 °C, respectively, using a Carbolite ELF11/6*B*/301 muffle, a γ power supply, and two stainless steel (AISI 304) electrodes separated by 4 cm.

### Material Characterization Techniques

Raman studies were carried out with an inVia Qontor confocal Raman microscope (Renishaw) equipped with a Renishaw Centrus 2957T2 detector and a 532 nm laser. 48-point spectra for each sample were measured and averaged, ensuring representative results for maps, high-resolution analyses, and depth measurements.

A Bruker D8 Advance model with a Bragg–Brentano 2θ configuration and Cu Kα radiation (*l* = 0.1542 nm) was used in Wide-Angle X-ray diffraction (WAXD) studies. All samples were characterized using a 2θ range of 20°–60° in steps of 0.02°, using a one-dimensional Lynx Eye detector.

Scanning electron microscopy (SEM) images were acquired by using a Zeiss Neon40 microscope equipped with an SEM GEMINI column. Pore size and porosity calculations using SEM images were performed by acquiring data from more than 10 different pore regions, ensuring sample representability.

High-Resolution Transmission Electron Microscopy (HR-TEM) images were acquired by using a JEOL 2010F microscope equipped with a field emission electron source operating at 200 kV. The point-to-point resolution was 0.19 nm, and the resolution between the lines was 0.14 nm. The samples were suspended in water (Milli-Q) solution using ultrasound, and then, one droplet was placed over a carbon-coated grid.

Electrochemical impedance spectroscopy (EIS) measurements were performed using a VIONIC potentiostat-galvanostat from Metrohm with a conductivity cell consisting of two stainless steel electrodes (AISI 304) isolated by a resin holder in the frequency range of 1 MHz–10 mHz and applying a 100 mV sinusoidal voltage. Cylindrical pellets were prepared for the tests, ensuring a complete electrode contact. Electrical Equivalent Circuits (EECs) were obtained by fitting the experimental data and obtaining a quadratic least-squares error (χ2) lower than 0.001.

### Continuous-Flow Reactions

Continuous-flow CO_2_ reactions were performed by using a commercial continuous-flow reactor (NovaLight CUBE 100, Peschl Ultraviolet GmbH, Germany). The reactor was designed to allow light irradiation, enabling coupling with different light sources, such as UV or solar lamps, for photocatalytic reactions. The reactor was also operated without an additional light source. All reactions were executed by introducing 1 mL of liquid water into the reactor, a wet CO_2_ flow (bubbled gas with Milli-Q water) of 50 mL/s, and a 120 °C temperature under different irradiation conditions. All the studied processes were repeated three times to ensure reproducibility. The UV lamp model used was GPH265T5*L*/4 (253.7 nm), while the solar lamp purchased from ABET Technologies (SunLite Solar simulator) was equipped with a 100 W xenon arc lamp. For the solar light-irradiated reactions, the lamp height was calibrated at the AM 1.5G earth’s surface level.

The reaction products were captured using a cold trap working at ∼5 °C. Condensed liquid products and catalyst adsorbed (after 1 h of reaction) products were analyzed by ^1^H NMR spectroscopy. For catalyst product desorption, a solution containing 100 mM HCl and 50 mM NaCl was used along with sonication. All ^1^H NMR spectra were acquired with a Bruker Ascend 400 NMR spectrometer operating at 400.1 MHz. Deuterated water and tetramethylsilane, as internal standards for chemical shifts, were added. Yields were calculated by peak area integration, acquiring 256 scans in all cases to ensure a proper signal-to-noise ratio. Accurate quantification was achieved by regular calibration using external references. Finally, to ensure the elimination of the influence of impurity carbon, blanks and controls (representative spectra are presented in Figure S4) were always performed before each reaction.

### Density Functional Theory (DFT) Studies

The quantum ESPRESSO (QE) v.7.2 package^47^ was used for all DFT calculations. The exchange correlation energy treatment employed the generalized gradient approximation (GGA) with the Perdew–Burke–Ernzerhof (PBE) formalism.^48^ Ultrasoft pseudopotentials (PP) from the standard solid-state pseudopotentials (SSSP Precision v 1.3.0)^49^ library were used for DoS, bands, and adsorption energy studies, while Projected Augmented Waves (PAW) were used for Bader charge analysis.^50^ The kinetic energy cutoff for the wave functions was set to 40 Ry, while for the charge density, it was set to 350 Ry. The temperature was simulated by applying a Fermi–Dirac function smearing scheme with broadening consisting of 2.5·10^–3^ Ry (393 K). Models for p-HAp were assembled following hexagonal HAp (space group symmetry P6_3_ /m) with experimental cell parameters *a* = *b* = 9.432 Å and *c* = 6.881 Å. For p-HAp (exact replica from the experimental catalyst) and p25-HAp, 50% and 25% hydroxyl group vacancies were generated on the lattice columns, respectively, as reported in previous studies^34^; meanwhile, the systems’ total charge was corrected according to the number of missing charges. The TSP process effects were modeled by reorienting the hydroxyl groups toward the *z*-axis and relaxing the position to minimize steric hindrances. The same procedure was carried out for c-HAp (exact replica from the experimental calcined material) and c25-HAp, with 50% and 25% vacancies, respectively. Hydroxyl-stabilized brushite (Bru) (partial surface replica from the experimental p-HAp/Bru) was modeled with experimental parameters as a monoclinic cell with *a* = 5.811 Å, *b* = 15.176 Å, and *c* = 6.234 Å and α = γ = 90 ° and β = 116.41° (space group symmetry I1a1).

The DoS and bands were calculated for p-HAp, c-HAp, Bru, p25-HAp, and c25-HAp using 8 × 8 × 8 Monkhorst–Pack k-point grids. For the band calculation, the optimal k-path was acquired using the SeeK-path tool.^50^ For the adsorption energy studies, according to the HR-TEM results, three slab supercells 2 × 1 × 2, 2 × 1 × 2, and 2 × 2 × 1 for the crystallographic planes (001), (101), and (121), respectively, were assembled (168 atoms each) with a 30 Å vacuum thickness. The γ point calculation was used to reduce the computational time due to the slab size. Additionally, for some energetically unstable binding sites, bond constraints have been employed to study the minimum energy achievable for specific binding sites. van der Waals Grimme-D3 correction implemented in Quantum ESPRESSO v.7.2 package was employed for all the simulations involving adsorption energy calculation, thus accounting for weak atomic interactions. The equation *E*_ads_ = *E*_slab+CO2_ – *E*_slab_ – *E*_CO2_, where the energies of the sole slab (*E*_slab_) and CO_2_ molecule (*E*_CO2_) are subtracted from the energy of the slab with adsorbed nitrogen (*E*_slab+CO2_), was used for the adsorption energy calculation. Charge Density Differences were calculated using the equation ρ = ρ_total_ – ρ_slab_ – ρ_CO2_, and the latter image representation employed the isosurface ρ = 0.0015 e/Å. Bader charge analysis was performed by extracting charges from QE computed charge densities using Bader Charge Analysis 1.05 version code from the Henkelman Group (University of Texas at Austin).^51^ Nudge Elastic Band (NEB) simulation for the CO_2_ dissociation pathway employed a Broyden optimization scheme with a total of 12 images, while the path error threshold was 0.1 eV/Å. The initial and final images were previously optimized, ensuring proper starting and ending states. Finally, it is worth highlighting that, according to the current state-of-the-art, no modeling of the magnetic parameters has been employed in the present study.

## Supplementary Material



## Notes

Discussion on the suitability of the computational methods used, key performance indicators (Table S1); ^1^H NMR chemical shifts of products (Table S2); band gaps from DFT calculations (Table S3); equivalent circuit data from EIS measurements (Table S4); binding site disposition (S1); further structural characterization, ^1^H NMR results and reaction blanks and controls (Figures S1–S6); suitability of the computational methods used (Figures S7–S9); UV–vis spectra for c-HAp (Figure S12); complementary computational calculations (Figures S10–S17; except S12) (PDF)
