# Supplementary material for: Lattice Engineering in Hydroxyapatite Enables Direct Photocatalytic Synthesis of C4 Products from CO2
Source: ACS Appl Mater Interfaces. 2025 Dec 16;17(52):70621–33. doi: 10.1021/acsami.5c19539 (PMC12766675; doi:10.1021/acsami.5c19539)
Supplement: Supplementary file 1 [file am5c19539_si_001.pdf]

SUPPORTING INFORMATION

Lattice Engineering in Hydroxyapatite Enables Direct Photo-Catalytic Synthesis of C_4 products from CO_2

Marc Arnau,[a,b,*] Isabel Teixidó,[a,b] Pau Turon,[c] Carlos Alemán[a,b,d,*] and Jordi Sans[a,b,d,*]

[a] *IMEM-BRT Group, Departament d'Enginyeria Química, EEBE, Universitat Politècnica de Catalunya - BarcelonaTech, C/ Eduard Maristany, 10-14, 08019, Barcelona, Spain.*

[b] *Barcelona Research Center in Multiscale Science and Engineering, Universitat Politècnica de Catalunya - BarcelonaTech, 08930 Barcelona, Spain.*

[c] *B. Braun Surgical, S.A.U. Carretera de Terrassa 121, 08191 Rubí (Barcelona), Spain*

[d] *Institute for Bioengineering of Catalonia (IBEC), The Barcelona Institute of Science and Technology, Baldiri Reixac 10-12, 08028 Barcelona, Spain.*

* marc.arnau.roca@upc.edu, carlos.aleman@upc.edu and jordi.sans.mila@upc.edu

Page	Content
S3	Discussion on the computational methods used.
S4	**Table S1.** Key Performance Indicators
S5	**Table S2.** ^{1}H-NMR chemical shifts of products.
S6	**Table S3.** Band gaps from DFT calculations.
S7	**Table S4.** Equivalent circuit data from EIS measurements.
S8	**Scheme 1.** Binding Site disposition
S9	**Figure S1.** High-Resolutions Raman maps acquired for surface analysis.
S10	**Figure S2.** HR-TEM micrograph from c-HAp sample.<br>**Figure S3.** Desorbed product yields from reactions.
S11	**Figure S4** Blank reactions ^{1}H-NMR spectra.
S12	**Figure S5.** Catalyst adsorbed products selectivities.
S13	**Figure S6.** Reaction products ^{1}H-NMR.<br>**Figure S7.** Comparsion between PBE and PBE0 computational methods.
S14	**Figure S8.** Comparison assuming charge compensation of the p-HAp system in the computational methods.<br>**Figure S9.** Tauc plot analysis of p-HAp.
S15	**Figure S10.** DoS complementary calculations.<br>**Figure S11**. Hydroxyl group distribution in different HAp supercells.
S16	**Figure S12.** UV-Vis spectra for c-HAp.
S17	**Figure S13.** Additional Band diagrams for remaining structures.
S18	**Figure S14.** Adsorption energy studies for p-HAp (101) plane.
S19	**Figure S15.** Adsorption energy studies for p-HAp (121) plane.
S20	**Figure S16.** Charge density difference for 5 atom CO_2 on (001) plane.
S21	**Figure S17.** Charge density difference for 5 atom CO_2 on (121) plane.

DISCUSSION ON THE SUITABILITY OF THE COMPUTATIONAL METHODS USED

Band structure calculation including trap states often requires special attention, as different computational methods and approximations to the problem might produce different results. Although hybrid funcionals may reproduce band structures with higher precision, it is primordial ensuring that the computational methods used better reproduces/approaches the real experimental systems. Accordingly, we have computed the band strcutrue of p-HAp sample considering: 1) Comparison between the band gap (B_g) obtained from the density of states (DoS) using hybrid funcionals with Norm Conversgin Pseudopotentials (PBE0) with pure generalized gradient approximation functional (PBE) using Ultra Soft Pseudopotentials (USPP), Figure S7; and 2) Assuming charge compensation (Figure S8). Finally, the B_g obtained have been compared with the experimental Bg value dervied from the Tauc plot analysis (Figure S9). As it can be seen, the use of hybrid functionals can be descarded due to the strong divergence with the experimental B_g value (7.31 and 4.36 eV, respectively). Furthermore,if charge compenstion (using PBE with USPP) is not assumed, the Bg is also realtively large (*i.e.,* 5.68 eV). Therefore, a PBE with USPP computational method has been used for the rest of the study. Nonetheless, note how the presence of the trap state is maintained for both methods, further supporting the conclusions of the study.

Table S1. Key performance indicators. In heterogeneous catalysis, the definition of Turnover Frequency (TOF) by IUPAC as "molecules reacting per active site in unit time" has been borrowed from enzymatic catalysis and, for that, it has been a source of confusion (*state of the art: S. Kozuch and J. M. L. Martin "Turing Over" Definitions in Catalytic Cycles. ACS Catal. 2021, 2 (12), 2787-2794*). Indeed, we could provide reaction TOFs, however, these values would be general and would not account for all the different sub reactions occurring during the products formation. That is, the TOF for the acetic acid production will differ from the 1,4-dioxane one and it is highly likely that they will depend on each other. Furthermore, as it has been reported in this study, p-HAp has multiple binding sites with different catalytic activity, thus, the TOF will also change according to these. Oppositely to the literature metal catalysts, our studied materials do not have an homogenous atomic surface as it could be the case for pure Au nanoparticles. Thus, due to the difficulty to quantify the sites in which each of the reactions take place in, TOFs become unreliable indicators for this specific study. Nevertheless, a general TOF has been calculated mainly based on the study of Kozuch et al. which proposes to base TOFs on the energetic barrier of a reaction, in our case, using the activation energy derived in a prior study (quoted in the manuscript as [41]).

**Catalyst**	**Irradiation**	**Carbon balance (%)**	**TOF (s^{-1})**
p-HAp	No	0.66911 ± 0.02537	13840.81 ± 636.68
	Solar Light	0.00941 ± 0.00009	
	UV Light	0.00258 ± 0.00003	
p-HAp/Bru	No	0.00421 ± 0.00018	
	Solar Light	0.00053 ± 0.00002	
	UV Light	0.00019 ± 0.00005	
	Irradiation	Carbon balance (%)	
	No	0.66911 ± 0.02537	

Solar Light	0.00941 ± 0.00009
UV Light	0.00258 ± 0.00003
No	0.00421 ± 0.00018
Solar Light	0.00053 ± 0.00002
UV Light	0.00019 ± 0.00005

Table S2. Chemical shifts associated to the obtained reaction products.

Product	^{1}H-NMR Chemical Shift (D_2O)
Radicals	0.16
Ethanol	1.18
Tert-Butanol	1.31
Isopropanol	1.41
Acetic Acid	2.05
Acetone	2.23
1,4-Dioxane	3.71
Formic Acid	8.45

Table S3. Direct and Indirect Band gap transitions for the different designed crystal lattice models. Second half corresponds to the Valence-Trap state band gap.

Cell Model	Direct Band Gap (eV)	Indirect Band Gap (eV)
HAp	5.150	5.112
c25-HAp	4.783	4.761
p25-HAp	4.452	4.446
c-HAp	4.171	4.154
p-HAp	5.039	4.984
Bru	5.400	5.398

	Direct Trap Band Gap (eV)	Indirect Trap Band Gap (eV)
c25-HAp	2.543	2.522
p25-HAp	2.277	2.276
c-HAp	1.747	1.730
p-HAp	2.880	2.850

Table S4. Equivalent Circuit parameters acquired and fitted through Electrochemical Impedance Spectroscopy for c-HAp, p-HAp and s-Bru samples.

EEC Model	c-HAp	p-HAp	s-Bru
R_b [MΩ cm^{-2}]	568.66	9.65	0.98
Error [%]	2.40	5.88	6.47
CPE_b [pF·cm^{-2}·s^{n-1}]	20.61	-	
Error [%]	2.93	-	
n_b	0.93	-	
Error [%]	0.29	-	
R_w [nF·cm^{-2}·$s^{-1/2}$]	2.44	-	
Error [%]	10.26	-	
R_γ [MΩ cm^{-2}]	-	114.48	923.86
Error [%]	-	0.88	1.52
CPE_γ [pF·cm^{-2}·s^{n-1}]	-	23.89	28.24
Error [%]	-	2.65	1.77
n_γ	-	0.96	0.91
Error [%]	-	0.26	0.27
$R_{w\gamma}$ [nF·cm^{-2}·$s^{-1/2}$]	-	7.11	89.07
Error [%]	-	2.54	3.48
C_b [pF·cm^{-2}]	-	20.70	21.74
Error [%]	-	3.14	4.56

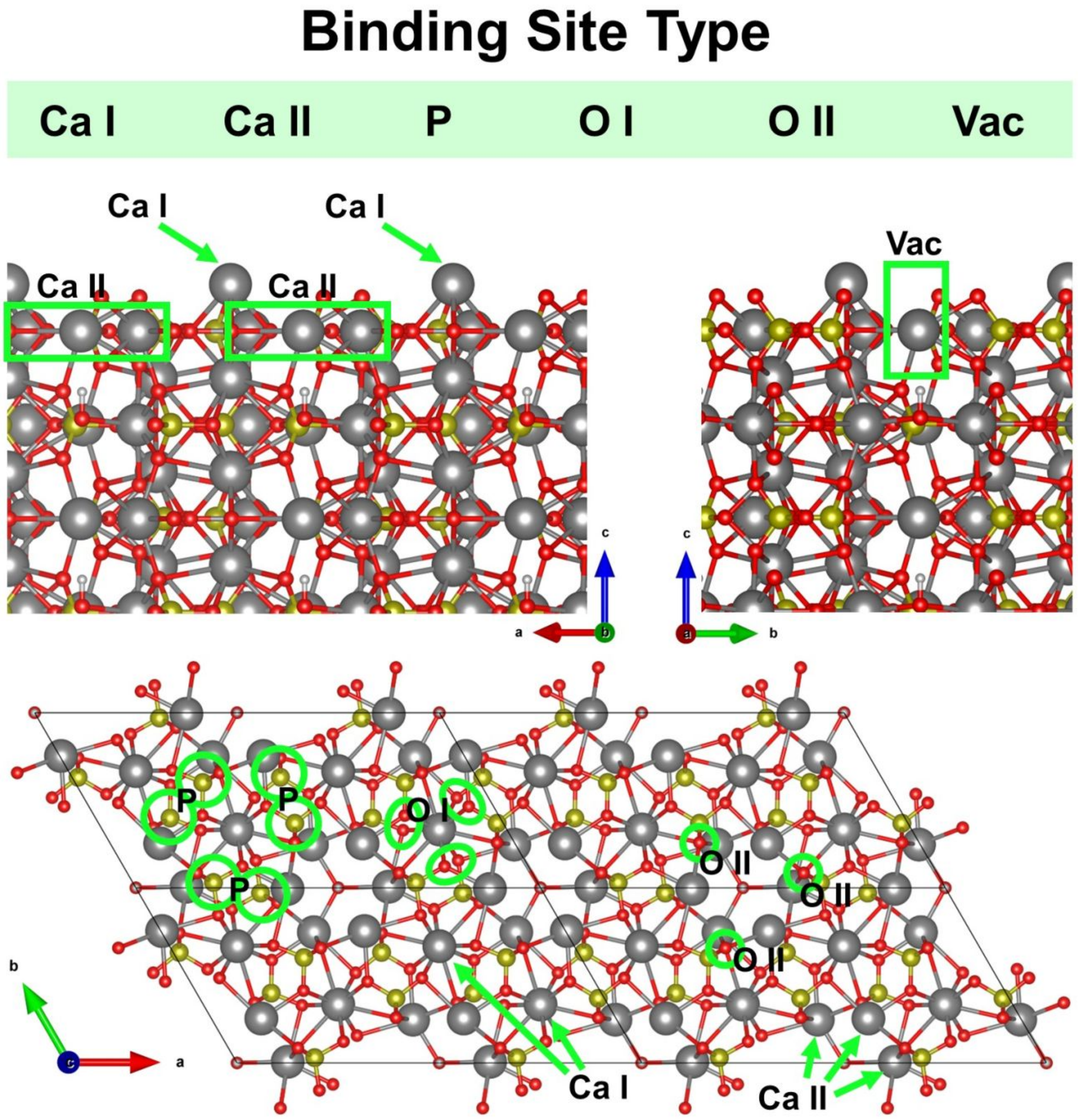

Scheme S1. p-HAp binding sites crystallographic postions. Slab with (001) plane was used for the illustrations.

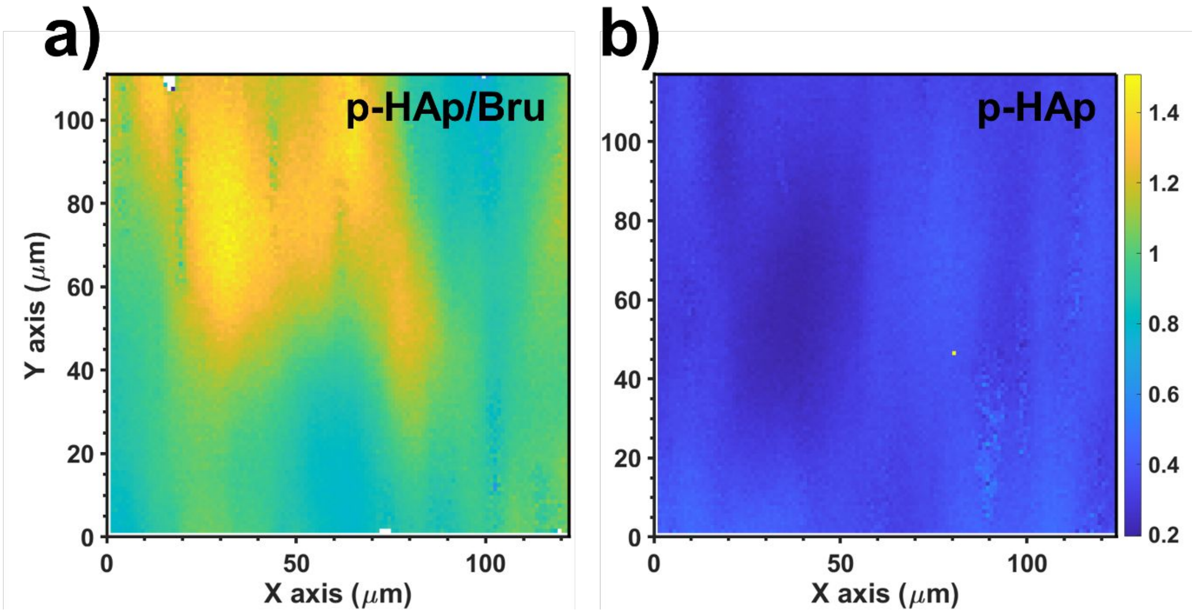

Figure S1. Raman high-resolutions maps where ratio between Bru 878 cm^{-1} peak intensity has been normalized with HAp 965 cm^{-1} active mode intensity, thus, being an indicator for Bru surface quantity localization. Measurements were acquired for a) p-HAp/Bru and b) p-HAp samples. It can be clearly seen that Bru amount increases significantly for p-HAp/Bru sample.

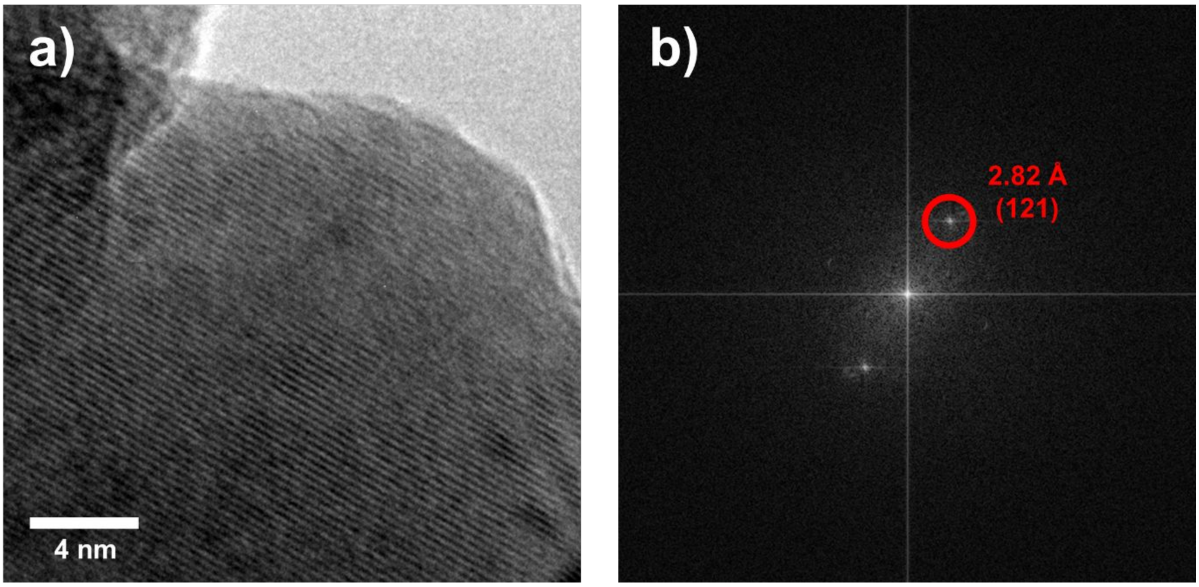

Figure S2. a) HR-TEM micrograph for the p-HAp/Bru sample displaying lattice fringes which have been associated to p-HAp as seen in b). b) Fast Fourier Transfor obtained from the area captured in a) where (121) lattice plane has been detected. No Bru cristallographic planes were detected.

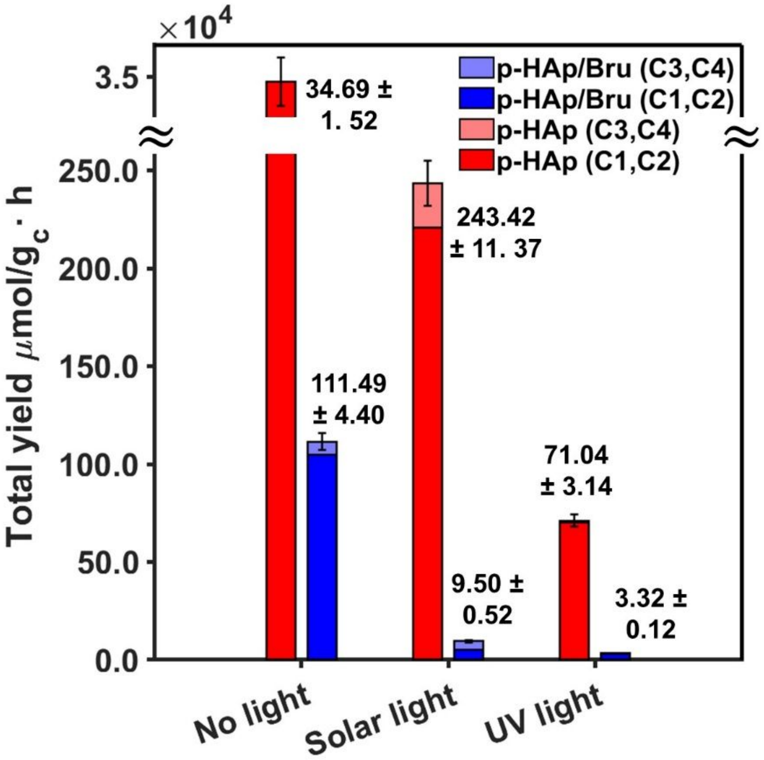

Figure S3. Desorbed products yield for p-HAp and p-HAp/Bru catalysts under different irradiation conditions. $C_1 - C_2$ and $C_3 - C_4$ products are differientated by color gradient.

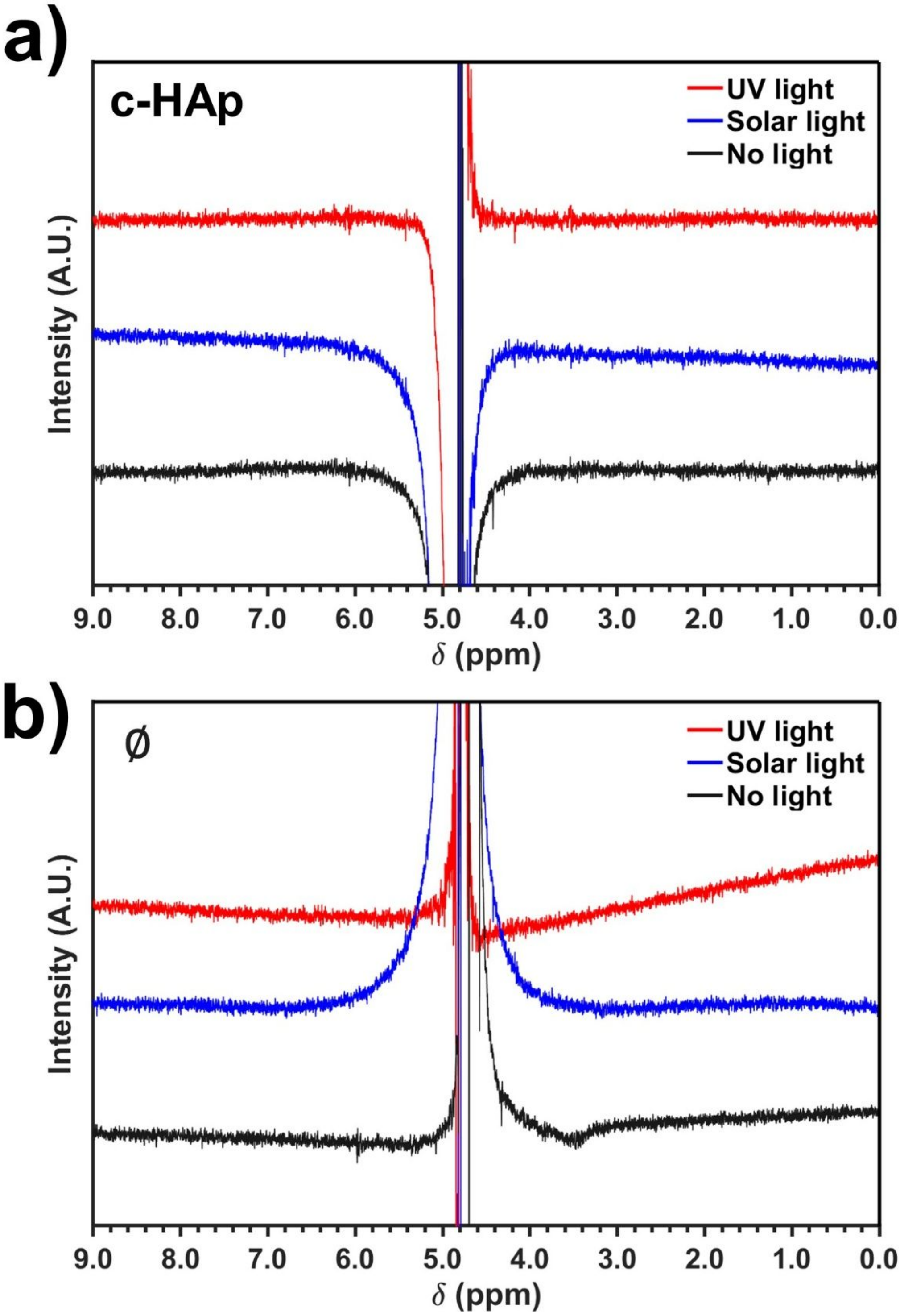

Figure S4. ^{1}H-NMR spectra collected for CO_2 continous-flow reactions of 60 min at 120 ºC using different irradiation conditions and a) catalyzed by c-HAp and b) in absence of catalyst/material (blank reactions). No desorbed products were detected in b).

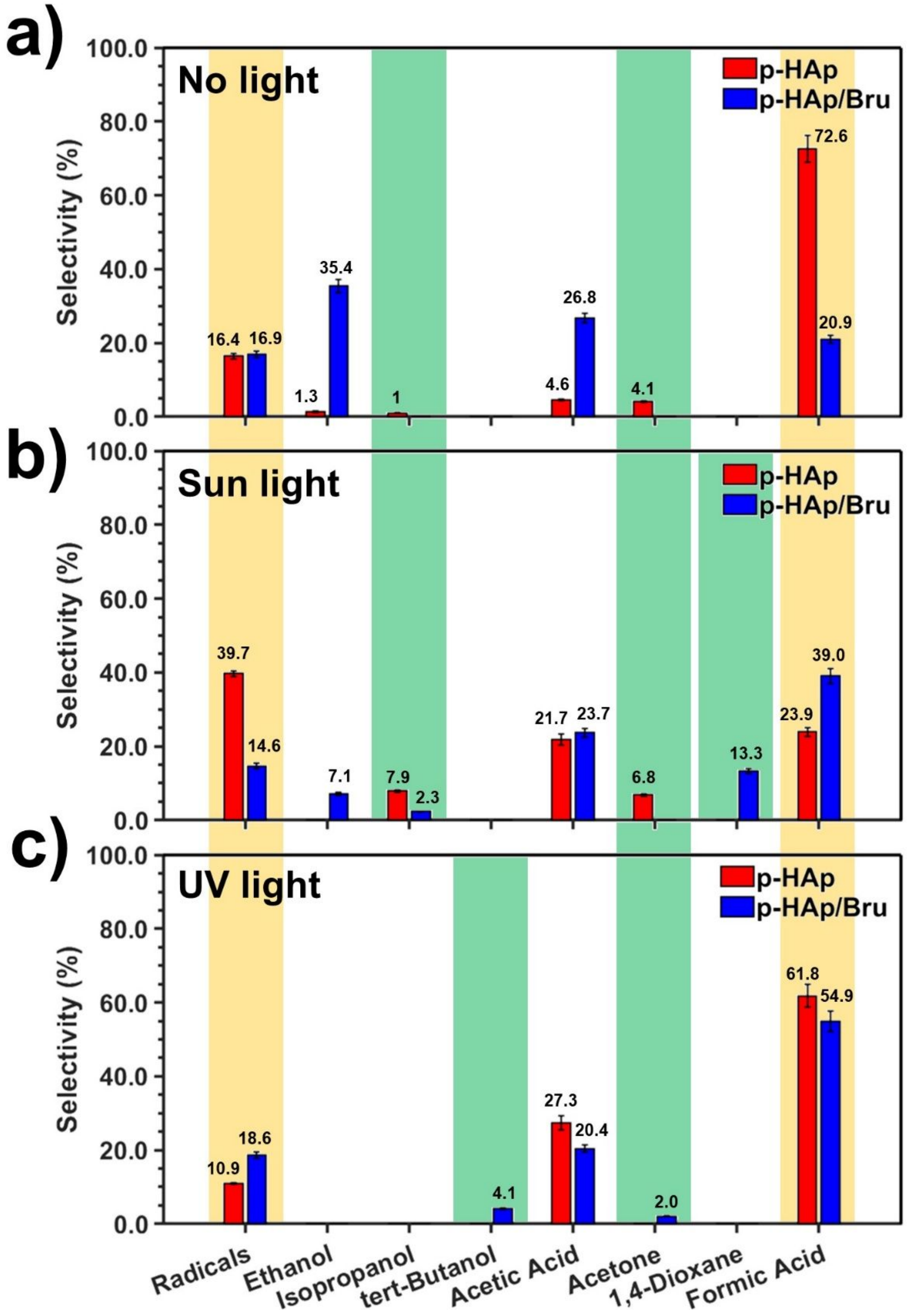

Figure S5. Selectivity percentages for each product generated under the three irradiation conditions, no light, solar and UV light (a), b) and c), respectively) for p-HAp (red) and p-HAp/Bru (blue). Gradient colored green has beenn used to mark C_3 – C_4 products while yellow for C_1 products.

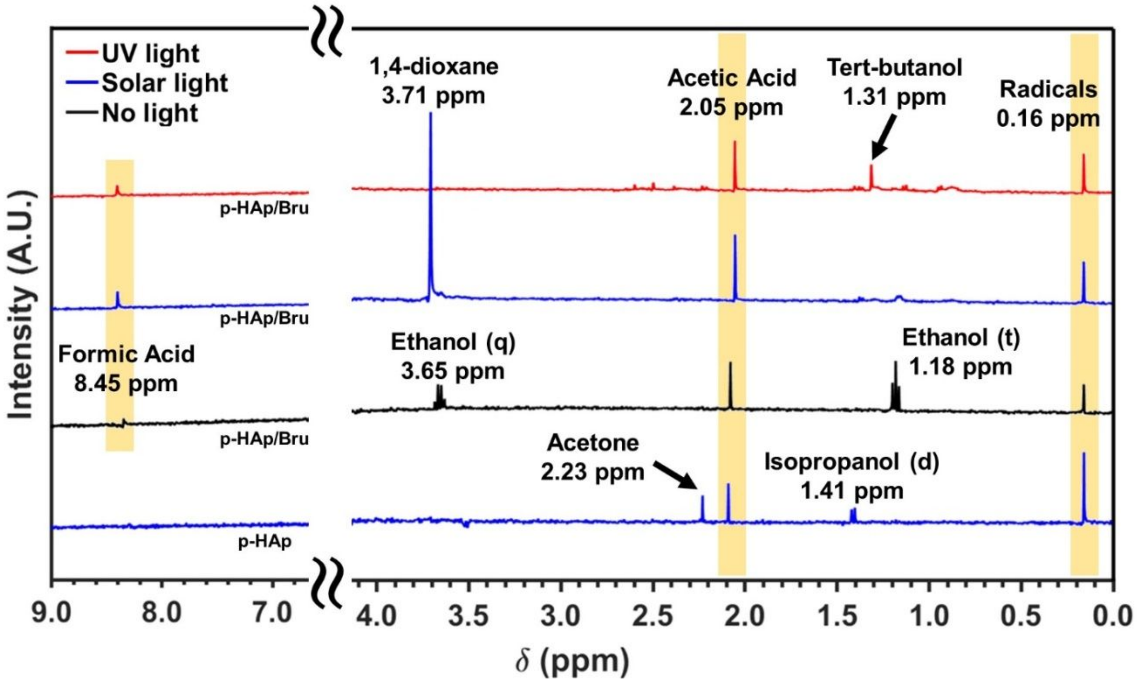

Figure S6. Representative ^{1}H-NMR spectra where all the products reported in the study can be appreciated with their respective chemical shifts.

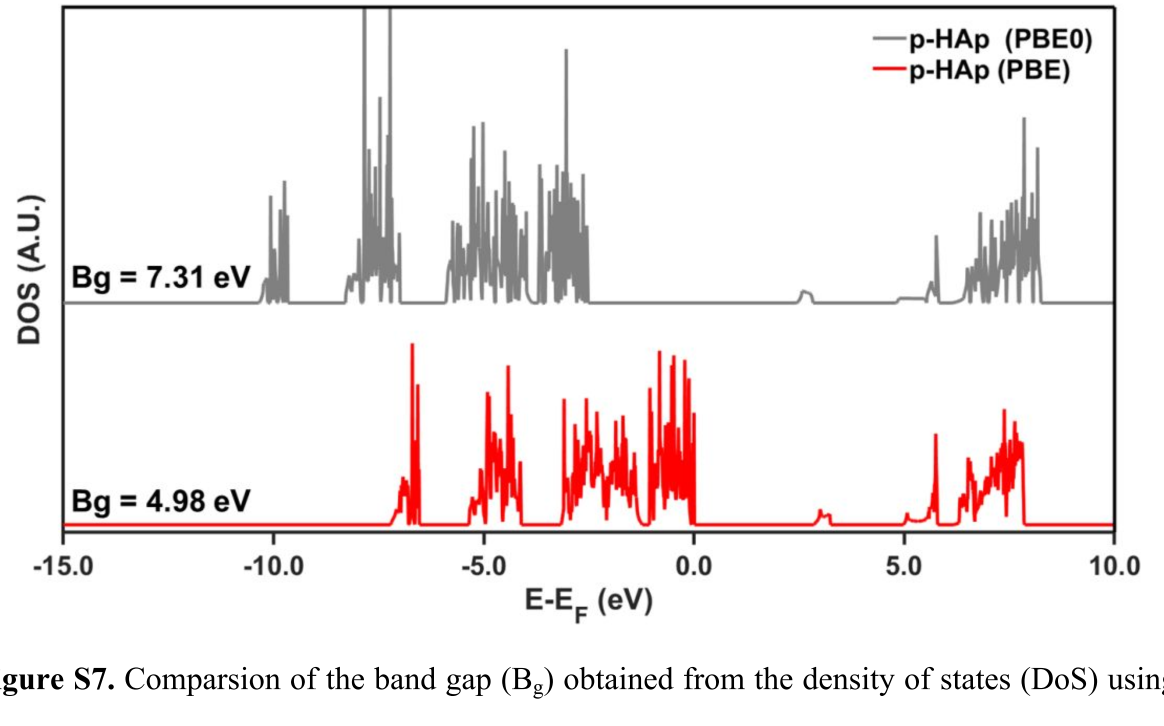

Figure S7. Comparsion of the band gap (B_g) obtained from the density of states (DoS) using hybrid funcionals with Norm Conversgin Pseudopotentials (PBE0) with pure generalized gradient approximation functional (PBE) using Ultra Soft Pseudopotentials (USPP).

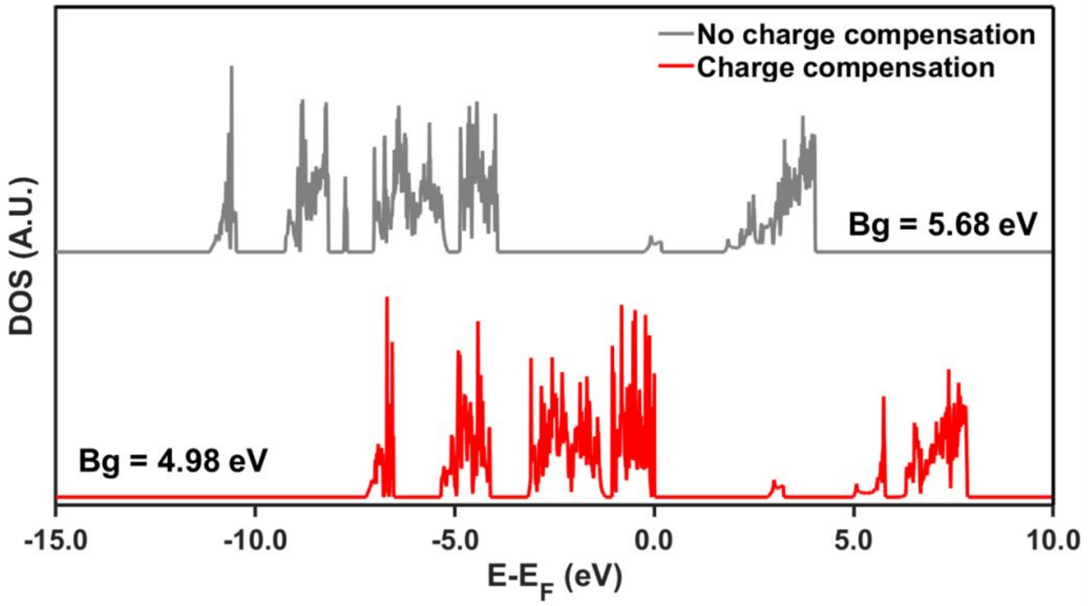

Figure S8. Comparison of the band gap (B_g) obtained from the density of states (DoS) assuming charge compensation. DFT methods: PBE functional with USSP.

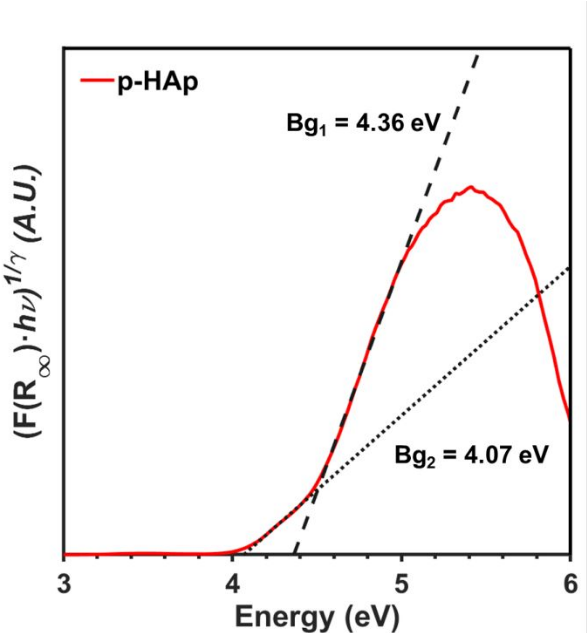

Figure S9. Tauc plot dervied from Figure 3 of the manuscript used to obtain the exerpeimental value of the band gap of the material.

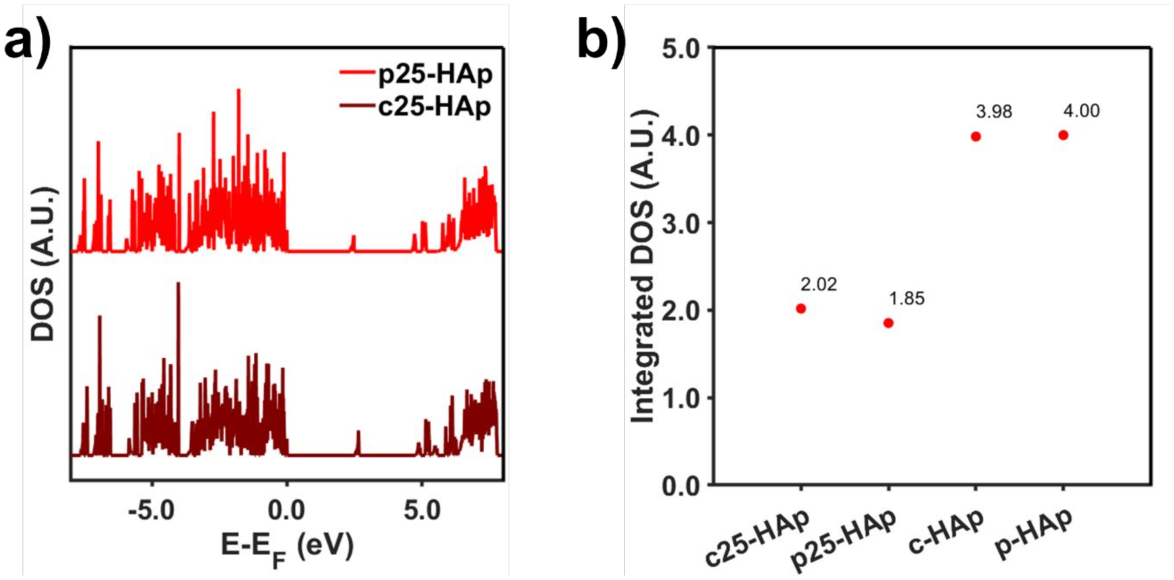

Figure S10. a) DoS for 25% vacancy generation samples p25-HAp and c25-HAp. Single generation of a trap state is obtained in both cases. b) Integrated DoS for modelled structres trap states.

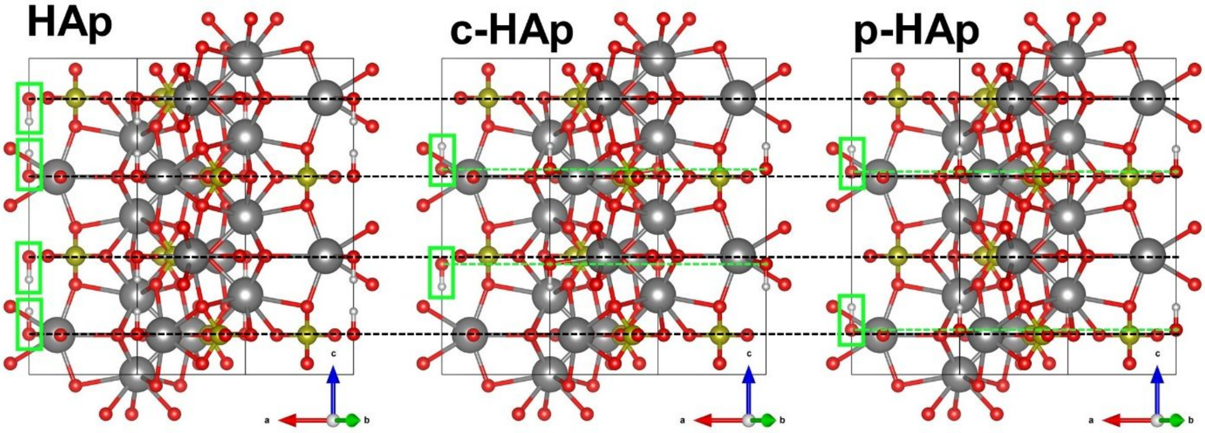

Figure S11. HAp, c-HAp and p-HAp supercells used for DoS and BG calculations. HAp hydroxyl groups positions have been marked with black dashed lines while their displacements in c-HAp and p-HAp with green dashed lines. Hydroxyl groups were relaxed in the *z*-axis (*c* axis) direction in all cases.

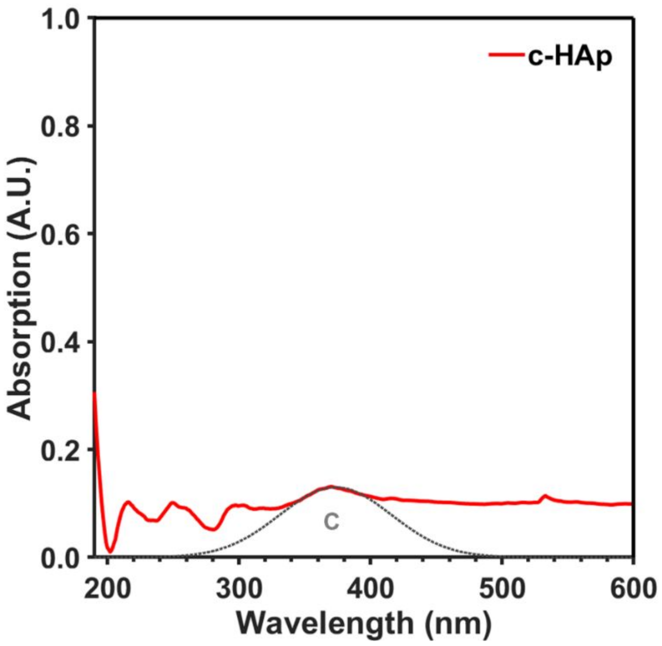

Figure S12. UV-Vis spectra for c-HAp sample where the C peak has been identified. Thus referring to a HAp contribution which is not altered by polarization.

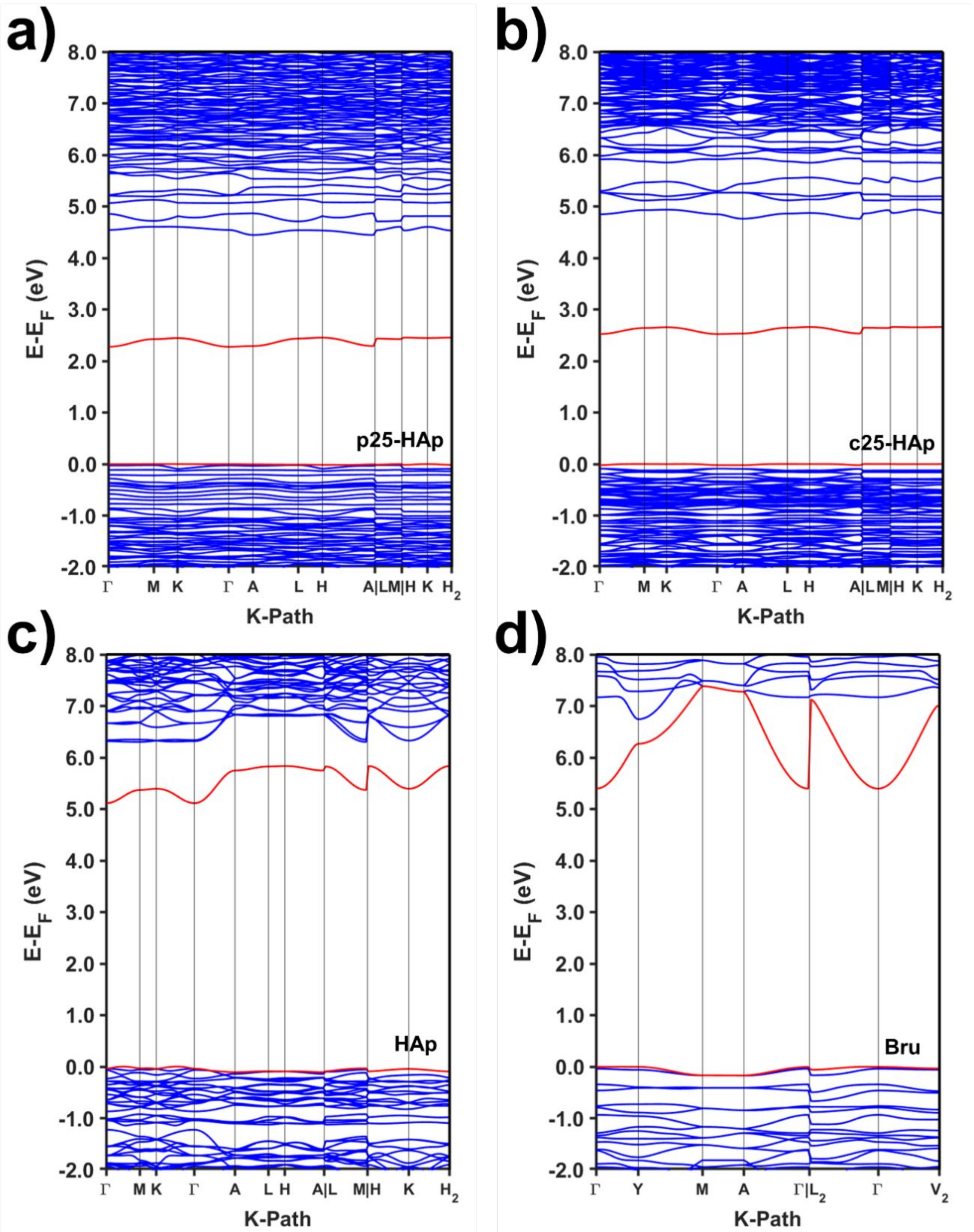

Figure S13. Band diagrams for p25-HAp, c25-HAp, HAp and Bru. Red color has been used to identify the last valence band and the first trap/conduction state band.

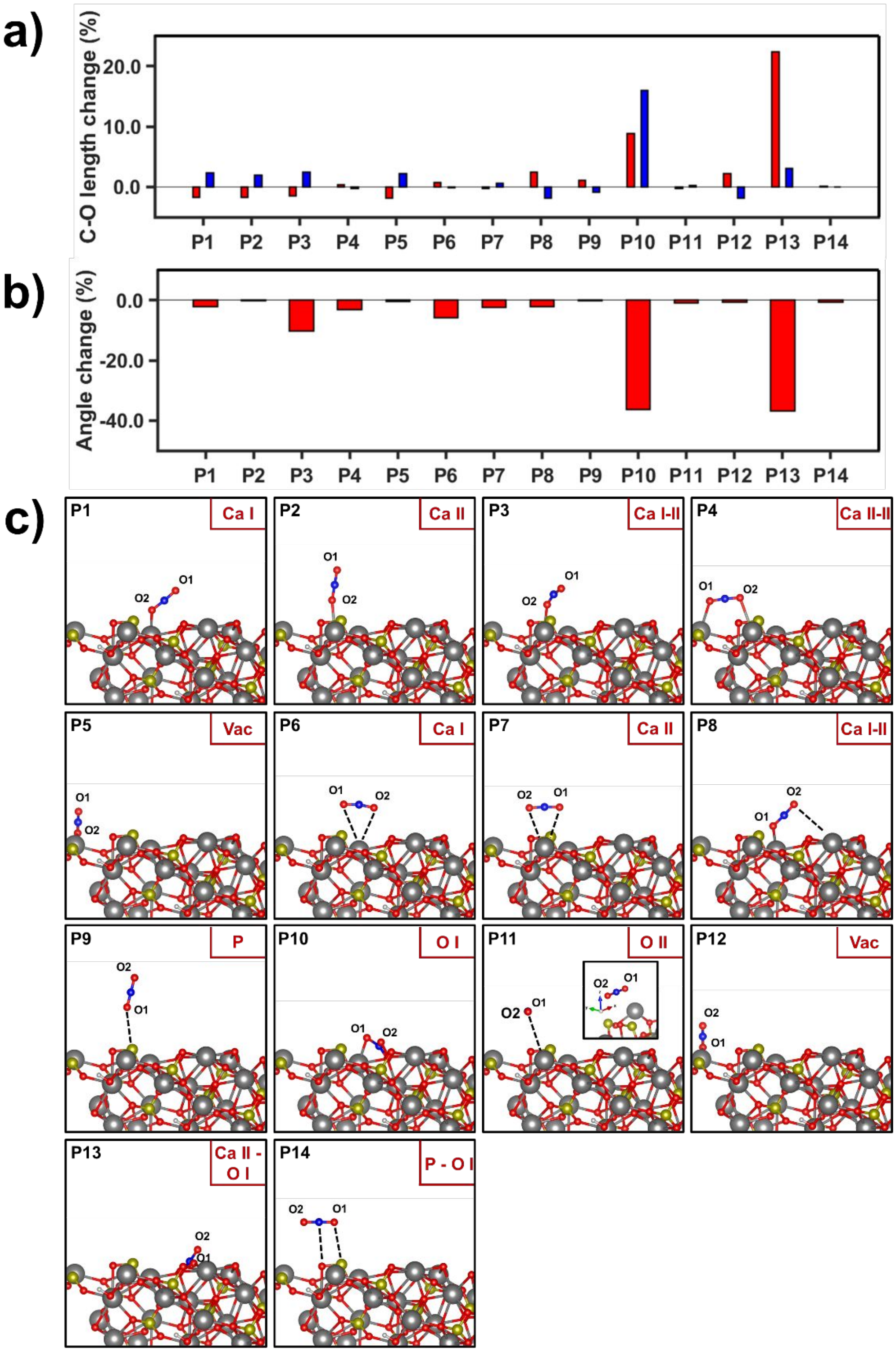

Figure S14. CO_2 adsorption energy studies for p-HAp (101) cristallographic plane. a) C – O bond length displacement % with respect to the original equilibrium value. b) CO_2 molecular bond angle change % with respect to equilibrium. c) 14 binding sites/positions studied where an adsorbed CO_2 molecule has relaxed to.

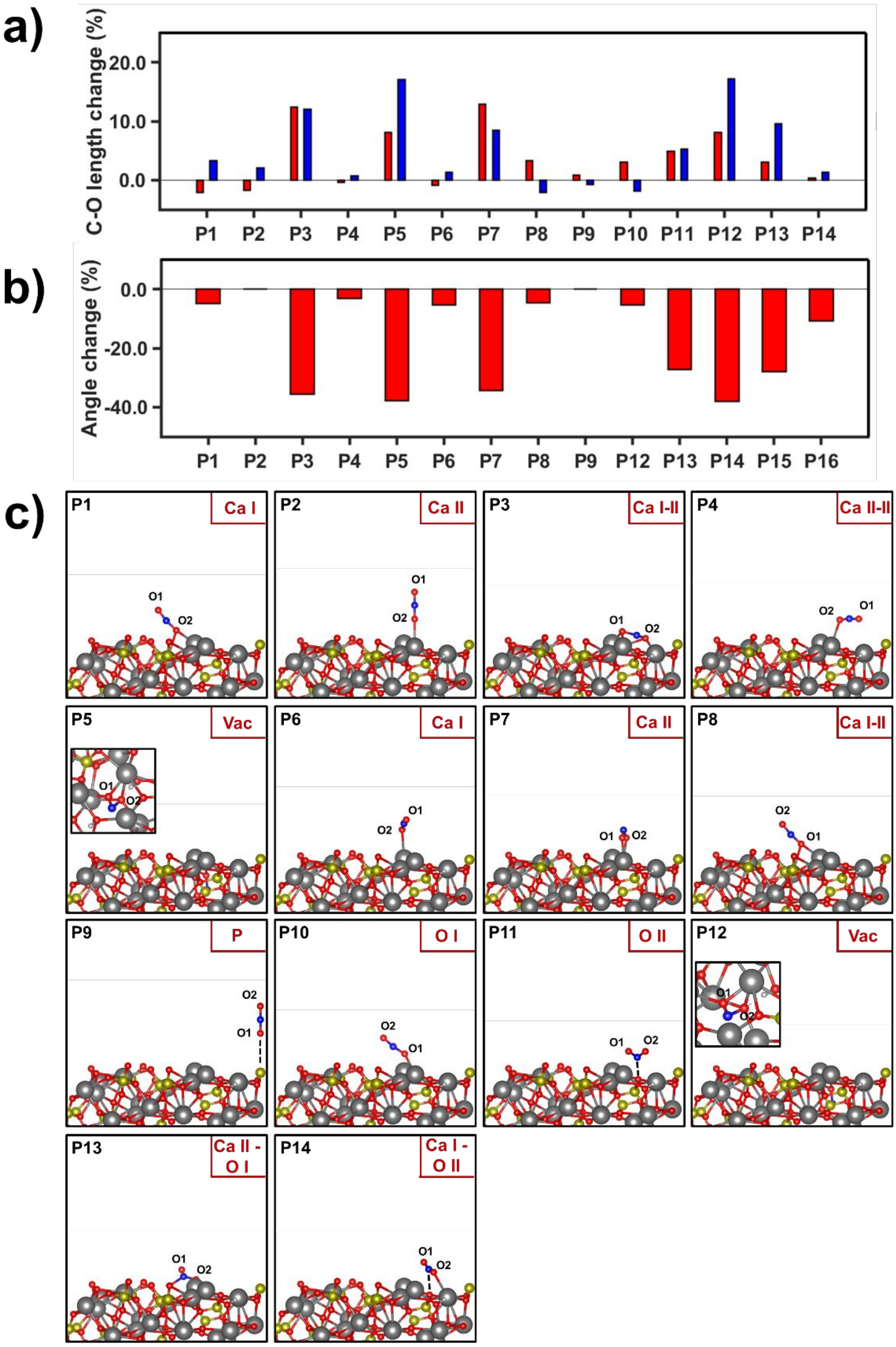

Figure S15. CO_2 adsorption energy studies for p-HAp (121) cristallographic plane. a) C – O bond length displacement % with respect to the original equilibrium value. b) CO_2 molecular bond angle change % with respect to equilibrium. c) 14 binding sites/positions studied where an adsorbed CO_2 molecule has relaxed to.

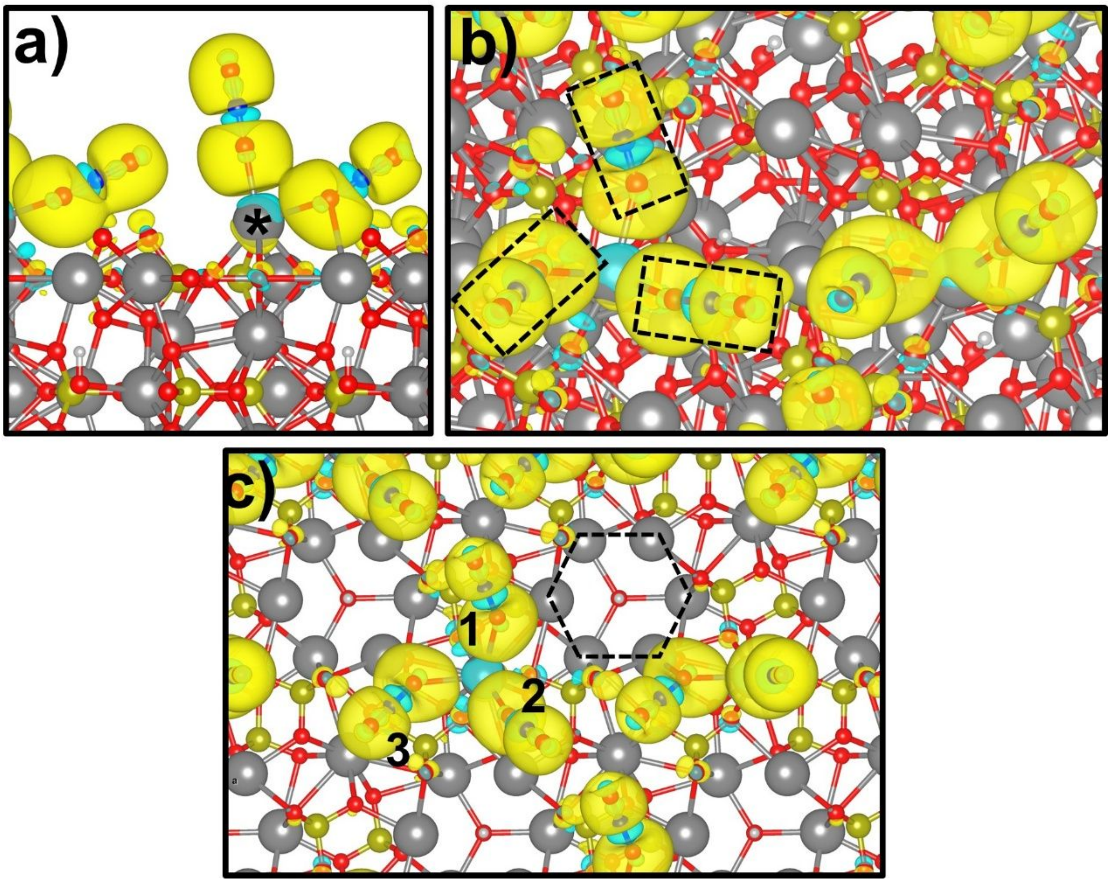

Figure S16. Relax DFT calculations for five CO_2 molecules on top of the (001) plane for p-HAp. a) Charge accumulation/depletion (yellow/blue) for the Ca I atoms. As mentioned in the main text, this highly charged centers attract more than one molecule when exposed upon this configuration. b) Different angle of a) illustrating charge accumulation regions. c) Relaxed molecules image where it is clearly observed that no relaxation occurs upon the hydroxyl vancacies/channels. Thus, further confirming that they do not play a direct role in the adsorption and act as bond cleaveage promoters.

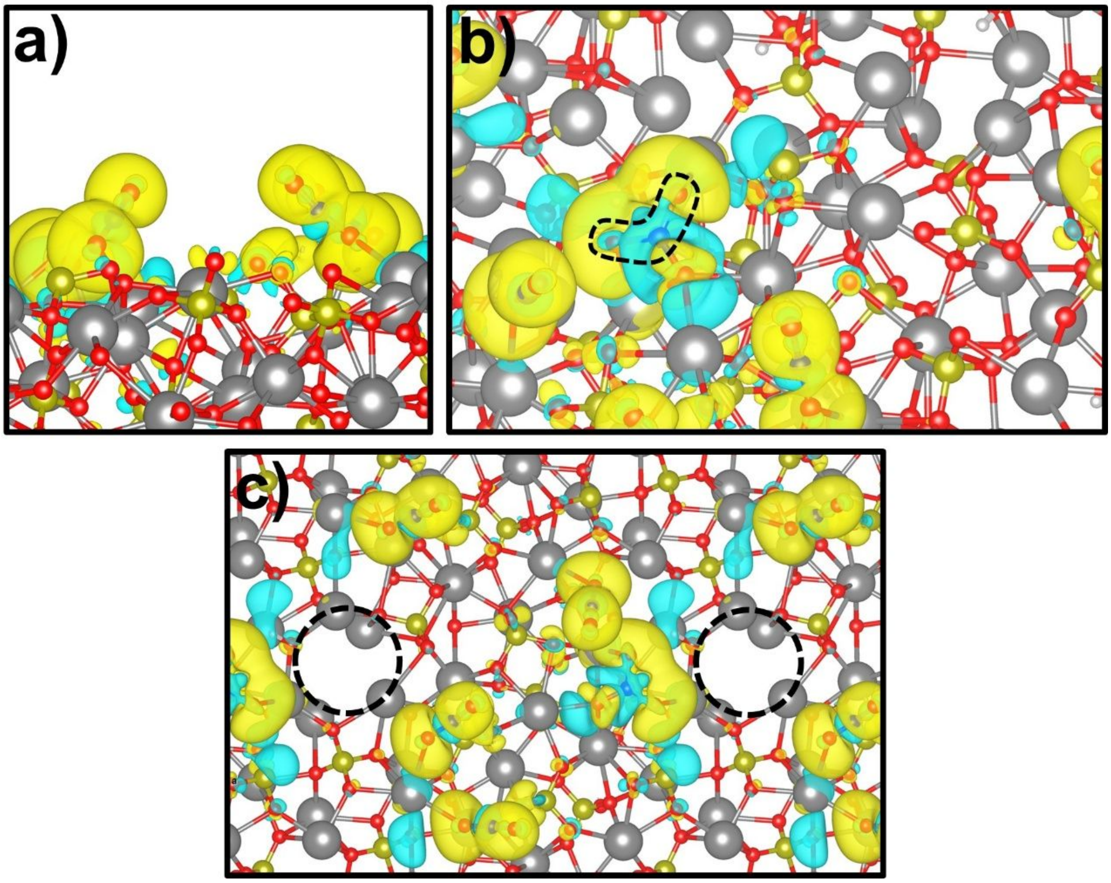

Figure S17. Relax DFT calculations for five CO_2 molecules on top of the (121) plane for p-HAp. a) Charge accumulation/depletion (yellow/blue). This plane promotes a more molecule-surface tight interaction. b) Different angle of a) illustrating charge accumulation regions. Some charge depletion regions not observed in the other planes appear and could be attributed to possible bond breaking promoters. c) As in Figure S13, no relaxation occurs upon the hydroxyl vancacies/channels.
